# Political uncertainty and multi-scale systemic risk spillovers in the cryptocurrency market: A STVAR-based network topology approach

**DOI:** 10.1371/journal.pone.0339488

**Published:** 2026-05-28

**Authors:** Zhecheng Wang, Xu Zhang, Wenting Xu, Xian Yang, Abdul Rauf

**Affiliations:** 1 School of Management Engineering, Nanjing University of Information Science and Technology, Nanjing, China; 2 School of Business, Hohai University, Nanjing, China; BRAC University, BANGLADESH

## Abstract

This study employs a smooth transition vector autoregressive model combined with a network topology approach to capture the nonlinear impact of political uncertainty on multi-scale systemic risk spillovers in cryptocurrency market. To reveal the risk characteristics at different time scales, we use wavelet packet decomposition to decompose the sequence into short-term, medium-term, and long-term components; We also use forecast error variance decomposition to quantify risk spillovers, in order to study the direction and intensity of risk spillovers between different cryptocurrencies. In terms of the responses of cryptocurrency systemic risks to political uncertainty shocks, we find significant asymmetries. The mid- and long-term risk components indicate that most cryptocurrencies exhibit a stronger response during periods of high political uncertainty than during low periods. Moreover, shocks during high political uncertainty periods enlarge the cross-cryptocurrency risk spillovers. Finally, Bitcoin, Ethereum and Monero are stable risk transmitters, while Peercoin and Namecoin are more vulnerable risk receivers. This paper provides some insights into the differential impact of political uncertainty on the stability and interconnectivity of various cryptocurrencies.

## 1. Introduction

In the aftermath of the 2008 global financial crisis, people began to favor an independent monetary system. Following the proposal of Nakamoto to create a decentralized electronic trading system, the cryptocurrency system was born. As more and more investors and speculators flood into the cryptocurrency market, cryptocurrencies exhibit characteristics of significant rises and falls, with extremely high volatility and susceptibility to external events. One study points out that due to the increased search interest in COVID-19, market volatility intensifies due to fear, and Bitcoin fails to become a safe haven under the impact of the pandemic [1]. As financial markets become increasingly integrated, one cryptocurrency may be influenced by others, resulting in strong interconnectedness among cryptocurrencies. Consequently, this situation has captured the attention of scientists, investors, and politicians. For instance, research suggests that although Bitcoin is initially regarded as a risk-averse currency, investors’ expectations regarding its risk-averse characteristics appear to be undermined by contagion risk [2]. In addition, in recent years, the intensification of major power competition and frequent regional conflicts exacerbate global political uncertainty. In this context, it is crucial to study the impact of political uncertainty on systemic risk spillover effects in the cryptocurrency market. Dagher *et al.* emphasize the importance of understanding how uncertainties caused by geopolitics and other factors affect the cryptocurrency market [3]. This paper mainly explores how political uncertainty affects the systemic risk spillover effect of cryptocurrency market and the risk spillover effect of different types of cryptocurrencies.

Our motivation for writing this article stems from two main points. Firstly, as more and more investors flock to the cryptocurrency market, which is characterized by high volatility and risk, the market gradually gains widespread attention. However, research on the impact of political uncertainty on its risk spillover may still be insufficient. On the other hand, the intensification of competition among major countries and frequent regional conflicts exacerbate the uncertainty of global politics. Recent events, such as those caused by the impact of the COVID-19, presidential election, the Russia Ukraine conflict, and the Israel Hamas conflict, bring about political uncertainty, and these events have an important impact on market behavior. Widespread instability (political) also repeatedly affects the cryptocurrency market [4]. As a new type of currency, cryptocurrency may avoid the risk of political and economic uncertainty to the traditional financial market. Some perspectives suggest that cryptocurrencies can serve as a hedge [5,6], while others believe that uncertainty can impact the cryptocurrency market, and negative geopolitical risks and other shocks can lead to the decline of Litecoin and Monero [7]. Many related studies focus on the returns of cryptocurrencies. This paper aims to explore from the perspective of risk. Therefore, the primary research objective of this paper is to investigate how political uncertainty affects the cryptocurrency market from the perspective of systemic risk spillovers, and to explore the role played by different types of cryptocurrencies in this process.

The research contribution of this paper is primarily manifested in two aspects. In terms of research subject, it differs from many existing studies. There is a lack of exploration into the impact of external political uncertainty on the cryptocurrency market. Although some studies may involve the relationship between economic policy uncertainty and cryptocurrencies, they either do not use variables specifically designed to measure political uncertainty or do not involve the risk spillover network of cryptocurrencies under political uncertainty. This paper, however, combines external political uncertainty shocks, multi-scale analysis, and the risk spillover network. In terms of the research methods, firstly, an analytical framework combining the STVAR model and network topology method is constructed to characterize the dynamic nonlinear impact of political uncertainty shocks on systemic risk spillovers in the cryptocurrency market. Secondly, an innovative multi-scale analysis method based on wavelet packet decomposition is applied to deeply analyze the performance of systematic risk at different time scales. This frequency analysis is crucial. Financial market participants have different behaviors, some prefer short-term speculation, while others prefer medium- and long-term investment. There may also be significant differences in risk transmission and perception at different time scales. Frequency analysis can more accurately reveal the internal structure of risk spillover. Although dynamic time-domain methods can see how the overall relationship changes over time, the differences in relationships at different time scales (frequencies) are fuzzy or average. In comparison to other time-frequency analysis methods, wavelet packet decomposition offers more flexible and refined frequency band partitioning. For instance, it excels in decomposing high-frequency components. Furthermore, this paper integrates forecast error variance decomposition and network topology methods to construct a visual risk spillover network, intuitively illustrating the systemically important cryptocurrencies and risk transmission under specific conditions.

This study mainly draws the following conclusions through empirical analysis: (1) In terms of the responses of cryptocurrency systemic risks to political uncertainty shocks, we find significant asymmetries. Mid- and long-term risk components identified indicate that most cryptocurrencies exhibit a stronger response during periods of high political uncertainty than during low periods. (2) The shocks in the period of high political uncertainty expand the risk spillover across cryptocurrencies. (3) BTC, ETH and XMR are stable risk transmitters, while PPC and NMC are more vulnerable risk receivers. This research is meaningful for some subjects. First of all, for investors, the research in this paper can make them clearly identify the different reactions of the cryptocurrency market and the risk contagion paths of various types of cryptocurrencies under different political uncertainties. And pay attention to the impact of political events on the market, and then optimize the asset allocation strategy under different political uncertainties. Secondly, for regulators, the network topology and multi-scale analysis provided in this paper can further identify systemically important cryptocurrencies and provide a basis for formulating optimized regulatory strategies or protection strategies. In addition, for academic researchers, the research framework of this paper combines multi-scale analysis, STVAR model and network topology method, which can provide a methodological reference for subsequent research on the impact of external shocks on complex financial systems.

## 2. Literature review

Many previous studies focus on measuring the risks and volatility of the cryptocurrency market [8–12], however, they have primarily utilized time domain analysis, which potentially obscures the details of dynamic variations. This cannot distinguish the risk Spillovers of different investment periods (such as short-term investment and long-term investment). From a theoretical perspective, investor term heterogeneity is an objective economic fact and has a certain impact on asset pricing. Information transmission in financial markets exhibits multi-scale characteristics, and investors with different investment horizons respond differently. Gençay *et al*. proposed that volatility differs across different time scales [13]. A state of high volatility in the long-term perspective does not necessarily imply a state of high volatility in the short-term perspective. These provide a theoretical foundation for multi-scale risk analysis. Therefore, other scholars have adopted wavelet analysis to analyze cryptocurrency market returns and volatilities across time scales [14–16]. But the application of this kind of method is not comprehensive enough, for example, by utilizing wavelet coherence, Qiao *et al*. examine how cryptocurrencies are interconnected and discover that the hedging effect between Bitcoin and other cryptocurrencies is even more pronounced at low frequencies [16]. However, it does not combine multi-scale spillovers with the impact of external factors.

As financial markets are fraught with uncertainty and instability, recent studies have focused on the strong link between uncertainty factors and market economics [4,17–19]. One study also classify the sources of uncertainty, including economic policy uncertainty (EPU), crude oil volatility index, etc., and point out that these uncertainties may have heterogeneous impacts on the hedging properties of financial assets [20]. From the perspective of behavioral finance, humans are not purely rational and are influenced by various factors such as social environment and emotions. Rising uncertainty can trigger investors’ “panic” or “cautious” emotions, thereby changing investors’ risk perception and investment decision-making behavior and this constitutes the theoretical basis for analyzing how uncertainty affects market conditions. Political conflicts and factionalism are influential factors that lead to uncertainty in a country’s economy [4], therefore, political uncertainty can impact financial markets to some extent. Junior *et al*. study the impact of the 2024 US presidential election on herd behavior in the cryptocurrency market and point out that political uncertainty triggers market segmentation in digital assets [21]. Cheng *et al*. report that spillovers from U.S. partisan conflict shocks to the euro area are more profound than those from U.S. policy uncertainty [18]. Meanwhile, Azzimonti investigates the effect of U.S. partisan conflict on private investment and finds a negative correlation between partisan conflict index (PCI) and total investment [22]. However, there is still a lack of research on the impact of political uncertainty on different time scales. In general, the multi-scale potential asymmetric impact of political uncertainty on the cryptocurrency market is a topic worthy of further discussion.

Diebold and Yılmaz further broaden the network topology approach, making network correlation a new perspective for exploring risk spillovers between cryptocurrencies [23]. This research direction is covered in the studies of Ji *et al*., Koutmos, Moratis, and Yi *et al*. [24–27]. Moratis (2020) finds that Bitcoin dominates the cryptocurrency risk spillovers but that size is not the only determinant affecting shocks [26]. These studies enable us to effectively identify whether one cryptocurrency is systemically important, However, these studies still have defects, they do not consider the impact of external factors，such as economy and Politics, and do not quantify the nonlinear impact of political risk on the network structure.

In some articles, the research involves cryptocurrency and economic policy uncertainty. A study points out that uncertainty can impact the cryptocurrency market, and shocks such as negative geopolitical risks can lead to a decline in Litecoin and Monero [7]. Foglia & Dai studied the spillover effects of economic policy uncertainty and cryptocurrency index by using time-varying parameter vector autoregressive model [28]. It is found that economic policy uncertainty has significant spillover effects across countries, and EPU can predict cryptocurrency uncertainty index to a certain extent. Colon *et al*. use panel OLS regression, quantile regression and other methods to study the impact of political and economic uncertainty on cryptocurrencies, and find that the cryptocurrency market has a positive response to economic policy uncertainty and geopolitical risks, but the response of cryptocurrencies to uncertainty is heterogeneous [29]. Wu *et al*. find that in a certain period, the increase of EPU bring higher returns to Bitcoin, Ethereum and Rippo [30]. Recently, He *et al*. and Simran and Sharma also study cryptocurrencies and economic policy uncertainty (EPU), pointing out that the growth of EPU have a negative impact on the long-term returns of Bitcoin and Ethereum [5,6]. Cryptocurrencies such as Bitcoin and Ethereum are not reliable hedging investments in the long term. They can effectively hedge EPU in the short term and play the role of hedging assets; but stable coins such as Tada coins show risk aversion in the long run. A study also points out that neither cryptocurrencies nor traditional safe-haven assets exhibit consistent hedging and safe-haven characteristics in the face of uncertainties such as EPU [20]. However, the above research mainly focuses on the selected indicator EPU, which represents economic policy uncertainty and cannot specifically represent political uncertainty. Moreover, these literature mainly focus on studying the impact of economic policy uncertainty and other factors on the return rate of cryptocurrencies, in order to explore the hedging or hedging properties of cryptocurrencies. Currently, there is still a lack of research on the risk spillover network of the cryptocurrency market, and this paper fills this gap.

The current research also solves the gaps in the previous literature in some unique ways. promotes the research on the risk spillovers of the cryptocurrency market, and makes a contribution to the existing knowledge system. Firstly, different from the linear network model in traditional research, this paper uses the smooth transition vector autoregressive (STVAR) model to construct a network to study the nonlinear impact of political uncertainty on the systemic risk spillovers of cryptocurrencies. Secondly, different from the traditional time domain analysis and wavelet decomposition method, we use the wavelet packet-based method to study the multi-scale systemic risk of the cryptocurrency market, which provides a more flexible and more refined frequency band division. We also combine this with the impact of external factors. In this way, we reveal the different effects of political shocks on the risk spillovers of the cryptocurrency market at different time scales. Third, since previous studies often regard cryptocurrencies as an isolated system, we innovate and combine external political shocks with network analysis to identify cryptocurrencies with systematic influence and the risk spillover effects of cryptocurrencies.

In addition, considering the above literature content and key gaps, especially ignoring the impact of political uncertainty, the lack of combination of external political impact and network analysis, we need to put forward some testable assumptions:

Although the existing literature study the relationship between economic policy uncertainty and cryptocurrency, on the one hand, the indicators selected in these studies do not specifically represent political uncertainty. We believe that external factors such as political uncertainty are factors that cannot be ignored in the study. Studies show that political conflict and factionalism are factors that contribute to the country’s economic uncertainty [4]. On the other hand, these literatures mainly focus on the impact of economic policy uncertainty on the return rate of cryptocurrency, and there are few studies on the dynamic response degree and risk spillover of cryptocurrency. In recent years, increasing global political uncertainty has a significant impact on financial markets, and cryptocurrencies are a highly volatile part of financial markets. Hypothesis 1 is proposed as follows:

**H1**: The impact of political uncertainty has an asymmetric effect on the response of cryptocurrencies.

Then, generally speaking, the outbreak of major political events increase the volatility, uncertainty and risk of the financial market, and the entire cryptocurrency market is likely to be no exception, so the overall risk spillovers of the market will be enhanced. Hypothesis 2 is proposed as follows:

**H2**: Shocks during high political uncertainty periods enlarge the cross-cryptocurrency risk spillovers.

Due to the lack of combination of external political impact and network analysis, we need to further study the cryptocurrency risk network. Moratis finds that bitcoin played a dominant role in cryptocurrency risk spillovers [26]. Furthermore, it is likely that large cryptocurrencies mainly act as risk transmitters, while small cryptocurrencies do the opposite. This statement should still hold when considering the impact of different political uncertainties. Hypothesis 3 is proposed as follows:

**H3**: Large cryptocurrencies are stable risk transmitters while small cryptocurrencies are more vulnerable risk receivers.

## 3. Methodology

### 3.1 STVAR model

To capture the nonlinear impact of political uncertainty on the spillover effects of systemic risk in cryptocurrencies, we refer to the research of Zhang et al. [31] and adopt the STVAR model. STVAR stands for Smooth Transition Vector Autoregressive model, which is constructed based on the Vector Autoregression model (VAR model) but allows model parameters to vary under different states. The STVAR model is based on the vector autoregressive model (VAR model). VAR model takes each endogenous variable in the system as a function of the lag value of all endogenous variables in the system to construct the model. The general form of the model is:


$$\begin{array}{c} Y_{t}=A_{\text{0}}+\sum_{\text{i}=1}^{\text{p}}A_{\text{p}}Y_{\text{t}-\text{i}}+\varepsilon_{\text{t}} \end{array}$$ (1)

where $Y_{t}$ is an endogenous variable vector, $A_{\text{0}}$ is a constant term vector, $A_{\text{p}}$ is a coefficient matrix. p is the lag order of the model, $\varepsilon_{\text{t}}$ is the perturbation vector of vector autoregressive model. We do not use the original VAR model, but on this basis, we add the logical transfer function to construct the STVAR model. The STVAR model is a variant of the vector autoregressive model. It introduces a smoothing mechanism that allows model parameters to change in different states to capture nonlinear characteristics in economic and financial time series.

The reason for choosing the STVAR model lies in the fact that the traditional linear VAR model struggles to depict the varying impacts of external shocks (such as political uncertainty) across different market states. Given the high volatility of the cryptocurrency market, which may be significantly influenced by external shocks, there may be notable differences in risk transmission and response to shocks during periods of high and low political uncertainty. The STVAR model can achieve a smooth transition of the regime, which is suitable for capturing the smooth situation of dynamic changes, and can also better explain the macro regime transition. It is more conducive to the test of the previously mentioned hypothesis H1, that is, to compare the response of cryptocurrencies to political uncertainty shocks in different states.

In this study, we use conditional value at risk (CoVaR) to illustrate the systemic risk of eight cryptocurrencies: Bitcoin, Ethereum, Litecoin, Monero, Ripple, Namecoin, Feathercoin, and Peercoin. For detailed theories and calculations of CoVaR, refer to Adrian and Brunnermeier [32]. In this study, the quantile regression method is used to measure systemic risk. Following Caggiano *et al.*, we model the selected cryptocurrencies with a STVAR model [33]:


$$\begin{array}{c} Y_{t}=\text{[}1-F\text{(}z_{t-1}\text{)]}\overset{p}{\underset{L}{\prod }}Y_{t-1}+F\text{(}z_{t-1}\text{)}\overset{p}{\underset{H}{\prod }}Y_{t-1}+\varepsilon_{t} \end{array}$$ (2)

$$\begin{array}{c} \varepsilon_{t}\sim \;N\text{(}0,\Omega \text{)} \end{array}$$ (3)

$$\begin{array}{c} F\text{(}z_{t}\text{)}=\{1+\text{exp[}-\gamma \text{(}z_{t}-c\text{)}{\text{]}\}}^{-1},\gamma >0,E\text{(}z_{t}\text{)}=0,Var\text{(}z_{t}\text{)}=1 \end{array}$$ (4)

where $Y_{t}$ represents a set of endogenous variables; $F\text{(}z_{t}\text{)}$ is a logistic transition function used to describe the probability of samples being classified into different levels of political uncertainty (low and high uncertainty); the parameter γ determines state conversion rapidity; $z_{t}$ is a state variable used for characterizing the political uncertainty; c is the threshold parameter identifying different political uncertainty conditions; $\overset{p}{\underset{L}{\prod }}$ and $\overset{p}{\underset{H}{\prod }}$ indicate the coefficient matrix of low and high political uncertainty periods, respectively; and $\varepsilon_{t}$ is the vector of reduced-form residuals following a normal distribution, with zero mean and a variance–covariance matrix Ω. This expression illustrates how the endogenous variable $Y_{t}$ depend on the previous period’s state and the coefficient matrices under these states.

In model (1), $Y_{t}$ is the endogenous variable vector including the state variable, $Y_{t}=\left[z_{t},\;BTC_{t},\;ETH_{t},\;LTC_{t},\;XMR_{t},\;XRP_{t},\;NMC_{t},\;FTC_{t},\;PPC_{t}\right]$, where $z_{t}$ is the state variable processed by filtering and moving average; and $BTC_{t}$, $ETH_{t}$, $LTC_{t}$, $XMR_{t}$, $XRP_{t}$, $NMC_{t}$, $FTC_{t}$, and $PPC_{t}$ respectively represent the CoVaR of Bitcoin, Ethereum, Litecoin, Monero, Ripple, Namecoin, Feathercoin, and Peercoin in a specific period. In addition, we estimate the parameters $\left\{\overset{p}{\underset{L}{\prod }},\overset{p}{\underset{H}{\prod }},\Omega ,\gamma ,c\right\}$ of models (1)–(3) using the conditional maximum likelihood method.

### 3.2 Wavelet packet decomposition

In order to study the multi-scale characteristics of systemic risk spillover, this study refers to the research of Zhang et al. [31] and uses wavelet packet decomposition to perform multi-scale decomposition on the systemic risk sequence of cryptocurrencies, which is a method based on discrete wavelet transform. There are different kinds of wavelet bases, the wavelet base we selected is Daubechies (dbN) wavelet, which has compact support and orthogonality.

Wavelet packet decomposition is a method that hierarchically decomposes time series data to capture information across different time scales. Wavelet packet decomposition decomposes the original time series into sub-bands of different frequencies through an filtering process. For a decomposition of level L, ${\text{2}}^{L}$ non-overlapping frequency bands are generated. Traditional risk analysis methods may fail to capture risk characteristics that manifest differently because of varying time scales. Although other dynamic methods (such as rolling window method) can see how the overall relationship changes with time, the differences in the relationship at different time scales (frequencies) are blurred or averaged. Wavelet packet analysis decomposes high-frequency parts that are not subdivided in wavelet analysis, thus making the decomposition match the signal spectrum and ultimately improving the time–frequency resolution [34]. The cryptocurrency time series at different scales can be obtained using the Mallat decomposition algorithm [35]:


$$\begin{array}{c} \overset{\text{(}2m\text{)}}{\underset{j-1,k}{d}}=\sum_{n\in Z}{\overset{―}{h}}_{n-2k}\overset{\text{(}m\text{)}}{\underset{j,n}{d}} \end{array}$$ (5)

$$\begin{array}{c} \overset{\text{(2}m+1\text{)}}{\underset{j-1,k}{d}}=\sum_{n\in Z}{\overset{―}{g}}_{n-2k}\overset{\text{(}m\text{)}}{\underset{j,n}{d}} \end{array}$$ (6)

where $\overset{\text{(}M\text{)}}{\underset{j-N,S_{i}}{d}}$ are the wavelet packet coefficients and N is the level of decomposition, which determines the depth and granularity of the decomposition. Wavelet packet decomposition divides a signal into multiple frequency bands (sub-bands). M is the sign of the obtained sub-bands $\text{(}M=1,2,\cdots {\text{2}}^{N}\text{)}$, where higher-indexed sub-bands typically contain high-frequency information while lower-indexed sub-bands usually contain low-frequency information. The wavelet packet decomposition produces $S_{i}={\text{2}}^{N}$ different sets of coefficients. Finally, $h_{n-2k}$ and $g_{n-2k}$ are dual operators.

We adopt a three-level Daubechies wavelet function to decompose our sample and arrange the third level based on frequency from low to high (i.e., D_1_–D_8_). Following Berger and Genay, who provide a detailed explanation of each time scale, we define D_1_ and D_2_, D_3_–D_5_, and D_6_–D_8_ as the scales capturing long-term trends, mid-run information, and short-run components, respectively [36].

### 3.3 Forecast error variance decomposition

Referring to Diebold and YiLmaz, we utilize forecast error variance decomposition (FEVD) to explore the cross-cryptocurrency risk spillover effects [23]. The rationale for selecting this method lies in its proposal to construct connectedness measures based on variance decomposition, which decomposes the forecast error variance of a variable into components attributable to shocks from other variables in the system, thereby enabling the quantification of connectedness among variables. In this study, this method allows us to decompose the forecast errors of endogenous variables over a given period into the contributions of each cryptocurrency in the system, thereby constructing an intuitive risk spillover network. This facilitates the identification of risk spillovers and receptions among different cryptocurrencies, revealing which cryptocurrencies act as risk transmitters or receivers. Compared with other approaches, this method can relatively accurately capture the direction and intensity of risk spillovers (as reflected in the “TO” and “FROM” measures) and also facilitates the analysis of total spillover effects. The predicted mean square error of N endogenous variables in the T period is decomposed into the impact contribution of each cryptocurrency. The FEVD matrix is presented in Table 1.

**Table 1:** Risk spillover effect matrix.

	$Y_{1}$	$Y_{2}$	…	$Y_{N}$	FROM
$Y_{\text{1}}$	$\overset{T}{\underset{11}{d}}$	$\overset{T}{\underset{12}{d}}$	…	$\overset{T}{\underset{1N}{d}}$	$\sum_{i=1}^{N}\overset{H}{\underset{1i}{d}},i\neq 1$
$Y_{\text{2}}$	$\overset{T}{\underset{21}{d}}$	$\overset{T}{\underset{22}{d}}$	…	$\overset{T}{\underset{2N}{d}}$	$\sum_{i=1}^{N}\overset{H}{\underset{2i}{d}},i\neq 2$
…	…	…	…	…	…
$Y_{N}$	$\overset{T}{\underset{N1}{d}}$	$\overset{T}{\underset{N2}{d}}$	…	$\overset{T}{\underset{NN}{d}}$	$\sum_{i=1}^{N}\overset{H}{\underset{Ni}{d}},i\neq N$
To	$\sum_{j=1}^{N}\overset{T}{\underset{j1}{d}},j\neq 1$	$\sum_{j=1}^{N}\overset{T}{\underset{j2}{d}},j\neq 2$	…	$\sum_{j=1}^{N}\overset{T}{\underset{jN}{d}},j\neq N$	$S^{T}$

In Table 1, $\overset{T}{\underset{ji}{d}}$ represents the proportion of changes $Y_{j}$ caused by the shock of an endogenous variable:


$$\begin{array}{c} \overset{T}{\underset{ji}{d}}=\frac{{\sum_{t=0}^{T-1}}_{\overset{\text{2}}{\underset{jit}{e}}}}{\sum_{t=0}^{T-1}trace\left(E_{t}{E^{\prime }}_{t}\right)} \end{array}$$ (7)

where ${\sum_{t=0}^{T-1}}_{\overset{\text{2}}{\underset{jit}{e}}}$ describes the prediction error variance of variable $Y_{j}$ caused by an endogenous variable $Y_{i}$, $E_{t}E^{\prime }$ is the covariance matrix of the prediction error, and $\sum_{t=0}^{T-1}trace\left(E_{t}{E^{\prime }}_{t}\right)$ represents the variance of the total forecast error.

FROM represents the risk spillovers of cryptocurrency $Y_{j}$ from other cryptocurrencies:


$$\begin{array}{c} \overset{T}{\underset{FROM,j\leftarrow ⬝}{S}}=\overset{N}{\underset{i=1}{\sum }}\overset{T}{\underset{ji}{d}},i\neq j \end{array}$$ (8)

TO represents the risk spillovers of cryptocurrency $Y_{i}$ to other cryptocurrencies:


$$\begin{array}{c} \overset{T}{\underset{TO,⬝\leftarrow i}{S}}=\overset{N}{\underset{j=1}{\sum }}\overset{H}{\underset{ji}{d}},i\neq j \end{array}$$ (9)

$S^{T}$ measures the total risk spillover effect among N cryptocurrencies:


$$\begin{array}{c} S^{T}=\frac{\sum_{j,i=1}^{N}\overset{T}{\underset{ji}{d}}}{N},i\neq j \end{array}$$ (10)

## 4. Data

This study focuses on Bitcoin, Ethereum, Litecoin, Monero, Ripple, Namecoin, Feathercoin, and Peercoin. The eight cryptocurrencies are abbreviated as BTC, ETH, LTC, XMR, XRP, NMC, FTC, and PPC hereafter. Our data cover monthly closing prices of the cryptocurrencies from September 2015 to May 2024. Data on cryptocurrency prices can be obtained from the coinmarketcap website (https://coinmarketcap.com/) or the investing website (https://cn.investing.com), the monthly data on CRIX comes from the CRIX-VCRIX website (https://thecrix.de/), and PCI data series is updated by The Federal Reserve Bank of Philadelphia monthly (https://www.philadelphiafed.org/surveys-and-data). The reason for using monthly data is that it needs to be matched with the variable measuring political uncertainty (PCI) in frequency. The political uncertainty has a long period of change, and the daily data is of little significance，monthly data can better capture the changing characteristics of political shocks. In addition, the monthly data has less noise and is relatively stable. According to the method described above, we decompose the time series of each set of cryptocurrency data into eight frequency bands (D1-D8), and define D1–D2, D3–D5 and D6–D8 as the scales to capture long-term trends, medium-term information and short-term components, respectively. The reason for choosing a three-tier decomposition (eight frequency bands) is that it can effectively capture information at different time scales in financial time series. Furthermore, if further subdivided, the number of frequency bands increase exponentially, leading to excessive fragmentation of information. In similar studies, Zhang *et al.* also adopt this method [2]. Under this approach, the high-frequency band (D6-D8) represents shorter cycles and can capture more subtle and rapid fluctuations; the mid-frequency band (D3-D5) represents the medium term, while the low-frequency band (D1-D2) represents longer cycles and can reflect coarse fluctuations and long-term changes. The smaller the frequency, the longer the period of change. The sample selection has the following characteristics: (1) Focus on systemically important cryptocurrencies. The research objects include BTC, ETH, LTC, and XRP, which account for a large share of the cryptocurrency market capital and have great attention in the market. (2) At the same time, pay attention to the heterogeneity of the research object, especially the supplement of small and medium-sized cryptocurrencies. For example, XMR is a privacy cryptocurrency, and the selection of NMC, FTC and PPC is of great significance for evaluating the performance of small and medium-sized cryptocurrencies under political shocks. The cryptocurrency index (CRIX) is an index that can depict the overall price dynamics of the cryptocurrency market; thus, we choose it as a market index to measure systemic risk. Studies show that CRIX is selected through model selection, which can well represent the cryptocurrency market and better track the market development [37].

The United States is an economic and political power. Its policy changes and partisan conflicts have a more obvious impact on global financial markets, including cryptocurrency markets. American politics is characterized by fierce partisan conflicts, which not only lead to serious political deadlock, but also lead to high political uncertainty. PCI can better quantify this political conflict and deadlock, which is also used in current scientific research, then we can more effectively analyze the impact of political uncertainty on the Systematic Risk Spillover of cryptocurrency Market. Thus, we choose the U.S. PCI proposed by Azzimonti as the state variable to characterize political uncertainty [22]. To accurately divide the political uncertainty periods, we first take the logarithm of the data and then we perform six moving averages on the data.

The descriptive statistics of the logarithmic returns of cryptocurrencies are presented in Table 2. The average return of ETH is the highest, with a value of 0.0755, but it comes with a higher price fluctuation (0.3415). BTC, with the largest market capitalization, is the most stable (0.2073). Notably, none of the eight cryptocurrencies has a skewness of 0; this result suggests that the logarithmic returns of the cryptocurrencies are not normally distributed.

**Table 2:** Descriptive statistics.

Variable	Mean	Maximum	Minimum	Std. Dev.	Skewness	Kurtosis
BTC	0.0541	0.5284	−0.4743	0.2073	−0.1127	−0.0154
ETH	0.0755	1.1515	−0.7688	0.3415	0.5704	1.1151
LTC	0.0322	0.9661	−0.5539	0.2683	0.6350	1.0634
XMR	0.0549	1.5506	−0.5859	0.3248	1.4608	4.9523
XRP	0.0399	2.2159	−1.1059	0.4316	2.0113	7.5022
NMC	0.0002	0.9947	−1.3115	0.3209	−0.2311	2.7089
FTC	0.0034	1.4279	−1.1030	0.4041	0.4900	1.2685
PPC	0.0035	0.8987	−0.6591	0.2654	0.7150	1.1974

Table 3 presents the correlation coefficients among eight selected cryptocurrencies. The table reveals that the correlation coefficients between cryptocurrencies are all positive, indicating that these cryptocurrencies generally exhibit similar trends and may be influenced by common political and economic factors. The range of these correlation coefficients is approximately 0.3 to 0.7, suggesting that there are still certain differences in the price trends of various cryptocurrencies. Among these cryptocurrencies, the highest correlation coefficient is observed between Bitcoin and Litecoin, while the lowest is between NMC and FTC.

**Table 3:** Correlation matrix.

Variable	BTC	ETH	LTC	XMR	XRP	NMC	FTC	PPC
BTC	1.0000	0.5579	0.7260	0.5399	0.4382	0.3704	0.4673	0.4689
ETH	0.5579	1.0000	0.6325	0.5390	0.6120	0.4650	0.3891	0.5749
LTC	0.7260	0.6325	1.0000	0.5540	0.6497	0.4994	0.4663	0.6002
XMR	0.5399	0.5390	0.5540	1.0000	0.4573	0.3181	0.3147	0.4489
XRP	0.4382	0.6120	0.6497	0.4573	1.0000	0.5337	0.4223	0.5284
NMC	0.3704	0.4650	0.4994	0.3181	0.5337	1.0000	0.3047	0.5421
FTC	0.4673	0.3891	0.4663	0.3147	0.4223	0.3047	1.0000	0.4783
PPC	0.4689	0.5749	0.6002	0.4489	0.5284	0.5421	0.4783	1.0000

## 5. Empirical results

### 5.1 Impulse response to political uncertainty shocks

– show the dynamic responses of each cryptocurrency to a one-standard-deviation increase in the PCI over different time scales. The red solid line and blue dashed line show the impulse responses in periods of low and high political uncertainty, respectively.

**Fig 1:**
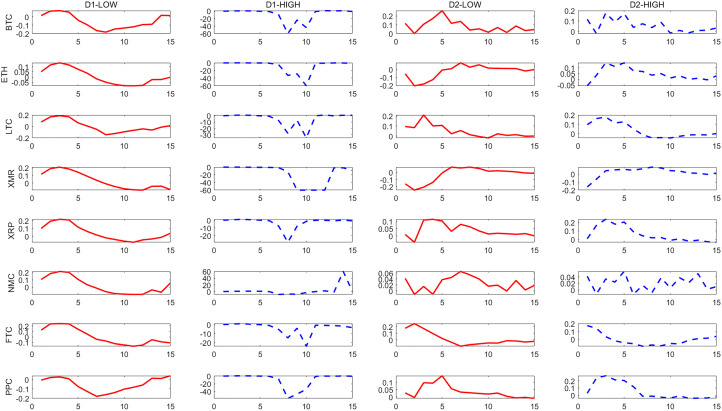
Effects of a shock to the U.S. PCI on cryptocurrencies at D_1_ and D_2_ scales. Note: Figs 1–4 show the impulse responses of eight cryptocurrencies to a one-standard-deviation increase in the Partisan Conflict Index (PCI) on D1-D8 (different time scales). The red solid line represents responses during periods of low political uncertainty, while the blue dashed line represents responses during periods of high political uncertainty. The horizontal axis denotes time periods after the shock, and the vertical axis denotes the magnitude of response.

**Fig 2:**
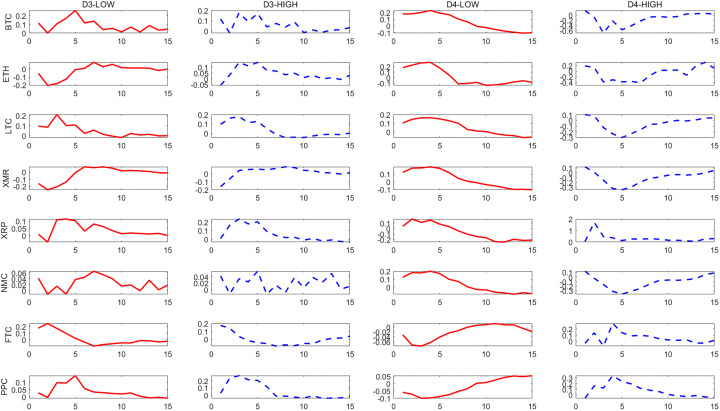
Effects of a shock to the U.S. PCI on cryptocurrencies at D_3_ and D_4_ scales.

**Fig 3:**
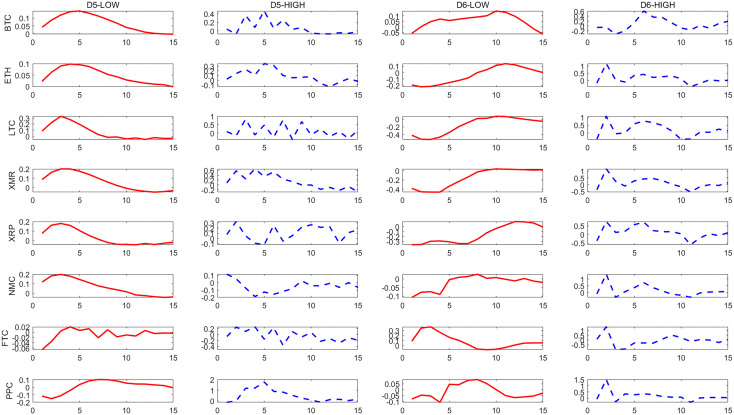
Effects of a shock to the U.S. PCI on cryptocurrencies at D_5_ and D_6_ scales.

**Fig 4:**
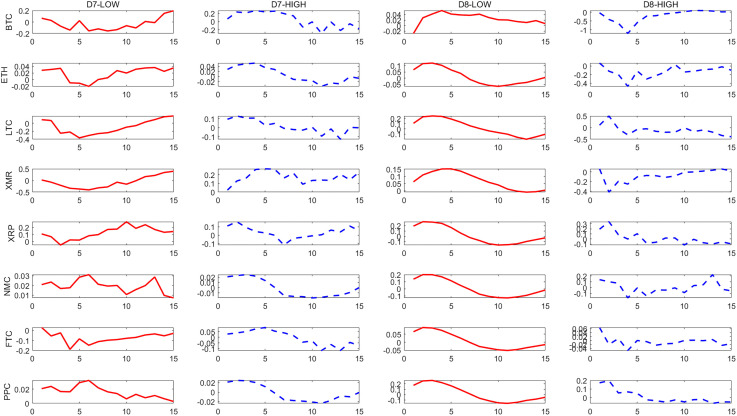
Effects of a shock to the U.S. PCI on cryptocurrencies at D_7_ and D_8_ scales.

The results indicate that there is a significant asymmetry in the impact of political uncertainty on cryptocurrencies, and overall, cryptocurrencies respond more strongly to high political uncertainty than to low political uncertainty. This asymmetry becomes more pronounced in the long run.

From the long-term scale (D1-D2), we find that the reaction of cryptocurrencies to the impact of political uncertainty is significantly asymmetric. On the whole, cryptocurrencies mainly respond to high political uncertainty more strongly than to low political uncertainty, among which BTC, ETH and PPC are relatively more prominent. This significance is more prominent at the D1 scale. This shows that cryptocurrencies respond more strongly to high political uncertainty in long-term investments and should be given more attention. It also shows that cryptocurrencies are not a good hedging tool on a long-term scale. Similar to the research results of He *et al*. and Simran and Sharma [5,6], they point out through different perspectives that most cryptocurrencies are not reliable safe-haven assets in the long term.

In contrast, this asymmetry is alleviated in the short and medium term. Specifically, at the D5 scale, the model predicts that in the case of high political uncertainty, BTC shows a greater positive response to the impact of 0.47, while in the periods of low political uncertainty, its response is only 0.15, and the amplification effect is more than three times. However, compared with the long-term, the difference in the response of these two political uncertainty conditions in the short and medium term narrows, especially in the short term, some currencies even show a strong response in the case of low political uncertainty. This shows that for short-term investments, it should be noted that the asymmetry of the impact of political uncertainty on cryptocurrencies has weakened relative to the long term, and short-term risk fluctuations may be dominated by other factors, which is similar to He *et al*. and Simran and Sharma [5,6]. They also point out that cryptocurrencies can more effectively hedge economic policy uncertainty in the short term and play a safe-haven asset role.

As mentioned previously, political uncertainty shocks have an asymmetric effect on cryptocurrencies. The identified medium- and long-term components show that most cryptocurrencies respond more strongly in high political uncertainty periods than in low political uncertainty periods. As one of the most important cryptocurrencies in the market, BTC is sensitive to the impact of political uncertainty.

Impulse response confirms that H1 holds, and the impact of political uncertainty has an asymmetric effect on the response of cryptocurrencies, which is mainly reflected in the fact that the response to high political uncertainty is stronger than that to low political uncertainty, and this effect is more obvious in the long-term scale.

### 5.2 Forecast error variance decomposition

Table 4 shows the multiscale FEVD of cryptocurrency in different periods of policy uncertainty. The percentages in the lower-right corner of each matrix indicate the total effect of risk spillovers. The results support the hypothesis H2 that shocks during high political uncertainty periods enlarge the cross-cryptocurrency risk spillovers, which is more pronounced in the long run (D1, D2 scale). For example, at the D2 scale, the total risk spillover effect in the periods of low political uncertainty is 74.68%, while the total risk spillover effect in the periods of high political uncertainty is 79.77%.

**Table 4:** Risk spillovers effect matrix of cryptocurrency in different policy uncertainty periods.

In low political uncertainty periods										In high political uncertainty periods									
Scale D_1_	BTC	ETH	LTC	XMR	XRP	NMC	FTC	PPC	FROM	Scale D_1_	BTC	ETH	LTC	XMR	XRP	NMC	FTC	PPC	FROM
BTC	32.85	7.28	3.64	5.44	3.26	5.37	4.01	3.10	67.15	BTC	15.19	38.26	5.55	10.55	7.09	1.62	1.54	7.31	84.81
ETH	16.32	17.86	3.44	7.15	2.93	3.73	6.34	3.10	82.14	ETH	12.80	39.47	5.52	10.52	7.70	1.58	1.73	7.07	60.53
LTC	26.59	7.75	4.42	6.41	2.79	4.75	4.28	3.11	95.58	LTC	13.14	38.33	6.20	10.48	7.39	1.58	1.55	7.52	93.80
XMR	11.15	14.88	2.64	13.60	2.23	3.88	5.21	2.83	86.40	XMR	13.65	38.53	4.07	12.93	6.64	1.64	1.66	5.67	87.07
XRP	25.95	7.46	2.58	6.39	6.28	3.95	4.32	3.37	93.72	XRP	12.59	38.19	5.58	10.22	8.85	1.51	1.57	7.57	91.15
NMC	12.58	8.79	2.86	7.75	2.71	12.57	3.20	3.38	87.43	NMC	8.93	40.83	5.53	9.72	7.32	3.58	1.54	8.01	96.42
FTC	18.05	10.67	3.08	9.83	2.89	5.19	6.70	3.53	93.30	FTC	12.09	37.99	5.26	10.63	7.50	1.69	2.71	7.64	97.29
PPC	26.00	9.80	4.25	7.11	2.54	4.57	4.16	8.75	91.25	PPC	13.28	37.64	6.02	12.16	7.04	1.65	1.56	7.80	92.20
TO	141.31	73.28	24.82	55.27	21.56	36.17	35.24	25.80	77.44	TO	92.79	307.40	42.13	87.16	56.82	12.89	12.78	57.12	78.14
**Scale D**_**2**_	BTC	ETH	LTC	XMR	XRP	NMC	FTC	PPC	FROM	**Scale D_2_**	BTC	ETH	LTC	XMR	XRP	NMC	FTC	PPC	FROM
BTC	25.97	6.14	6.48	4.51	15.16	2.80	4.93	2.00	74.03	BTC	16.42	4.68	5.07	4.55	8.79	5.98	8.72	3.12	83.58
ETH	3.39	26.21	2.98	5.23	6.22	3.13	6.21	2.14	73.79	ETH	2.22	10.81	2.00	5.67	5.55	6.58	9.63	2.54	89.19
LTC	7.15	8.50	16.97	5.70	11.33	4.21	7.38	2.09	83.03	LTC	3.76	6.60	9.68	5.77	7.91	7.25	12.34	2.79	90.32
XMR	16.52	6.97	6.20	12.35	15.60	2.93	4.92	1.83	87.65	XMR	10.16	5.70	6.48	8.11	9.59	6.41	10.21	2.16	91.89
XRP	3.43	18.33	9.90	6.48	15.10	3.96	6.27	1.20	84.90	XRP	3.53	8.20	5.11	6.95	9.48	7.70	13.62	2.59	90.52
NMC	4.55	7.22	8.88	6.84	12.70	10.28	8.62	2.27	89.72	NMC	2.36	6.52	5.57	7.04	10.09	8.06	12.12	2.67	91.94
FTC	8.59	11.19	3.69	6.64	7.53	3.87	13.22	2.03	86.78	FTC	4.17	8.05	2.75	5.77	6.56	5.85	13.71	2.64	86.29
PPC	4.51	7.59	6.12	5.96	9.14	4.76	10.69	7.75	92.25	PPC	2.73	6.42	3.81	5.96	7.52	6.26	14.55	5.79	94.21
TO	50.52	69.61	47.75	47.94	84.08	28.34	53.57	15.02	74.68	TO	30.85	49.60	32.63	48.85	60.95	51.42	89.60	21.68	79.77
**Scale D**_**3**_	BTC	ETH	LTC	XMR	XRP	NMC	FTC	PPC	FROM	**Scale D3**	BTC	ETH	LTC	XMR	XRP	NMC	FTC	PPC	FROM
BTC	51.14	4.40	3.41	4.76	1.35	2.46	3.46	9.26	48.86	BTC	48.78	11.20	1.56	6.99	2.56	1.35	3.73	6.88	51.22
ETH	17.64	32.35	2.37	4.07	1.93	3.68	3.18	7.88	67.65	ETH	16.52	37.44	0.83	4.57	2.19	5.35	2.53	8.75	62.56
LTC	28.25	8.63	9.79	6.99	2.13	3.29	3.35	5.05	90.21	LTC	21.67	13.64	6.38	11.28	2.72	3.33	3.15	5.23	93.62
XMR	15.34	6.62	5.16	12.56	2.39	4.98	1.69	5.67	87.44	XMR	17.85	12.70	2.13	19.22	2.90	2.42	3.50	6.74	80.78
XRP	26.44	9.37	8.19	6.96	5.86	3.25	3.21	6.27	94.14	XRP	21.03	14.87	4.93	10.65	6.76	3.64	3.04	6.77	93.24
NMC	35.85	4.66	4.91	11.95	2.46	12.23	3.74	5.33	87.77	NMC	31.87	11.01	3.24	14.90	4.12	11.36	2.96	5.58	88.64
FTC	15.02	4.83	3.13	5.01	2.24	5.00	5.44	5.53	94.56	FTC	16.17	12.41	1.13	7.18	1.94	2.31	11.19	4.80	88.81
PPC	16.58	9.87	7.16	3.98	2.24	3.57	4.58	25.24	74.76	PPC	15.89	15.12	4.45	6.47	4.14	4.11	4.65	16.07	83.93
TO	169.43	53.05	37.13	48.37	16.84	31.90	24.87	51.16	71.71	TO	155.64	103.04	19.32	69.86	22.21	25.24	28.19	51.38	71.42
**Scale D**_**4**_	BTC	ETH	LTC	XMR	XRP	NMC	FTC	PPC	FROM	**Scale D_4_**	BTC	ETH	LTC	XMR	XRP	NMC	FTC	PPC	FROM
BTC	28.35	3.19	3.49	13.59	10.73	1.74	5.26	1.76	71.65	BTC	13.56	1.30	4.85	53.31	3.20	2.78	3.70	2.30	86.44
ETH	12.84	28.96	2.40	12.91	9.67	2.34	3.39	3.84	71.04	ETH	7.01	7.64	5.21	53.47	3.49	2.92	4.67	2.19	92.36
LTC	8.21	2.66	9.79	12.20	12.83	1.59	4.19	3.67	90.21	LTC	7.26	1.11	7.87	51.06	4.13	3.34	4.55	1.91	92.13
XMR	6.24	3.34	3.45	12.56	12.40	1.82	4.06	1.94	87.44	XMR	5.18	0.95	4.42	54.97	2.92	3.31	4.41	1.83	45.03
XRP	6.05	4.95	2.51	11.83	38.12	1.20	3.67	10.61	61.88	XRP	5.90	2.08	5.32	52.93	9.59	2.07	5.05	1.84	90.41
NMC	6.83	4.13	3.62	11.32	10.09	2.04	4.07	1.64	97.96	NMC	5.72	1.07	4.64	53.01	2.84	3.45	4.16	2.00	96.55
FTC	7.41	7.06	5.79	30.84	8.31	1.50	10.86	8.58	89.14	FTC	3.93	1.52	4.55	62.57	3.14	2.85	7.90	1.84	92.10
PPC	8.68	5.14	4.06	11.59	13.36	2.93	4.19	16.88	83.12	PPC	7.69	2.37	4.58	48.30	4.95	4.56	4.21	6.30	93.70
TO	63.16	34.18	28.77	115.75	89.03	14.93	32.99	33.59	72.49	TO	48.12	11.40	38.00	428.43	27.62	25.17	35.06	15.86	76.53
**Scale D**_**5**_	BTC	ETH	LTC	XMR	XRP	NMC	FTC	PPC	FROM	**Scale D_5_**	BTC	ETH	LTC	XMR	XRP	NMC	FTC	PPC	FROM
BTC	30.35	2.17	1.40	2.63	4.75	2.86	5.94	3.67	69.65	BTC	20.78	1.04	1.29	9.05	2.95	6.82	15.93	17.74	79.22
ETH	20.01	14.28	3.18	3.24	2.15	4.36	6.28	5.87	85.72	ETH	17.08	6.50	1.53	6.18	2.07	5.70	16.87	19.91	93.50
LTC	7.76	13.09	15.97	2.78	2.16	2.34	5.15	4.51	84.03	LTC	18.08	4.47	6.15	5.93	1.62	3.78	16.60	15.06	93.85
XMR	10.05	3.84	1.48	21.78	1.65	2.85	6.25	4.81	78.22	XMR	18.60	2.03	1.17	9.53	1.50	3.90	13.43	13.67	90.47
XRP	11.09	6.10	2.79	4.01	18.69	3.01	5.95	5.18	81.31	XRP	13.15	2.91	1.84	5.60	6.17	4.11	18.23	19.42	93.83
NMC	3.74	3.22	1.65	3.26	1.51	18.28	7.67	4.00	81.72	NMC	6.84	1.15	1.90	7.86	1.54	10.53	24.52	19.54	89.47
FTC	7.78	7.86	3.16	14.70	2.33	7.06	20.65	4.19	79.35	FTC	21.33	2.90	1.90	7.48	1.94	4.66	15.97	14.30	84.03
PPC	15.66	2.54	2.00	7.66	3.13	9.28	10.32	18.43	81.57	PPC	11.25	0.96	1.99	11.30	2.56	6.37	20.99	23.91	76.09
TO	81.35	41.88	17.55	40.94	19.83	35.81	53.11	37.12	71.29	TO	112.87	16.97	12.90	58.75	15.50	39.65	144.80	141.72	77.83
**Scale D**_**6**_	BTC	ETH	LTC	XMR	XRP	NMC	FTC	PPC	FROM	**Scale D_6_**	BTC	ETH	LTC	XMR	XRP	NMC	FTC	PPC	FROM
BTC	43.91	7.27	3.65	4.78	2.47	5.43	2.38	5.26	56.09	BTC	22.87	11.58	10.27	16.08	1.48	7.53	2.17	2.09	77.13
ETH	25.95	13.98	3.14	6.08	3.34	6.30	3.42	3.09	86.02	ETH	16.26	14.65	9.06	18.48	1.59	8.59	2.64	2.45	85.35
LTC	13.48	5.97	5.98	5.60	2.67	9.75	1.90	4.96	94.02	LTC	10.51	12.64	11.37	18.84	1.69	7.81	2.58	2.14	88.63
XMR	8.49	8.18	3.59	15.89	2.43	6.55	3.46	3.05	84.11	XMR	10.08	13.05	10.69	22.42	1.46	6.88	2.77	2.10	77.58
XRP	8.19	4.92	4.60	6.65	16.34	7.57	2.26	4.54	83.66	XRP	9.81	11.85	10.96	18.06	6.62	6.14	2.58	2.17	93.38
NMC	24.72	9.55	5.14	6.02	1.92	17.53	2.10	6.36	82.47	NMC	11.94	14.25	10.00	19.26	1.49	15.11	2.62	2.99	84.89
FTC	5.67	5.84	3.22	6.37	5.70	4.53	32.21	4.01	67.79	FTC	7.09	13.18	9.00	20.42	2.48	4.98	13.94	2.13	86.06
PPC	26.48	7.36	10.04	6.85	2.23	8.56	2.18	9.83	90.17	PPC	13.34	13.60	10.85	18.04	1.36	12.91	2.89	4.41	95.59
TO	114.91	54.19	37.08	48.23	22.85	56.78	21.22	34.75	71.59	TO	83.17	102.25	82.03	148.24	12.97	61.59	21.39	17.89	76.51
**Scale D**_**7**_	BTC	ETH	LTC	XMR	XRP	NMC	FTC	PPC	FROM	**Scale D7**	BTC	ETH	LTC	XMR	XRP	NMC	FTC	PPC	FROM
BTC	29.66	8.53	6.10	6.40	11.60	10.11	1.36	5.16	70.34	BTC	29.58	3.83	4.18	8.66	7.54	3.48	1.27	3.90	70.42
ETH	13.78	15.09	6.15	6.90	8.68	9.02	2.43	4.72	84.91	ETH	5.47	17.20	5.94	5.47	4.79	3.20	2.90	1.76	82.80
LTC	10.08	20.76	9.07	6.39	11.03	11.48	3.08	4.28	90.93	LTC	4.70	24.60	9.14	9.01	6.60	4.32	4.62	1.63	90.86
XMR	19.93	9.04	8.25	12.71	13.01	11.11	1.42	5.28	87.29	XMR	18.34	5.45	5.15	16.94	11.34	4.65	1.77	2.41	83.06
XRP	14.91	10.82	8.79	7.23	16.70	9.15	2.16	4.22	83.30	XRP	9.81	8.15	6.84	6.20	23.15	2.86	1.98	1.42	76.85
NMC	14.89	8.71	6.22	5.14	7.08	9.63	1.96	5.41	90.37	NMC	2.47	4.22	5.11	5.90	10.10	3.42	1.77	1.61	96.58
FTC	15.08	6.85	9.73	13.20	9.16	9.01	10.77	3.86	89.23	FTC	8.23	4.97	5.75	18.79	8.99	5.55	15.88	2.81	84.12
PPC	14.89	9.01	5.80	5.02	6.52	9.66	1.94	5.53	94.47	PPC	2.48	4.17	4.91	5.95	10.14	3.85	1.86	1.72	98.28
TO	118.75	82.87	56.80	55.16	73.76	79.42	16.26	38.24	76.76	TO	54.00	59.79	42.72	65.90	69.70	32.04	17.92	17.15	75.89
**Scale D**_**8**_	BTC	ETH	LTC	XMR	XRP	NMC	FTC	PPC	FROM	**Scale D8**	BTC	ETH	LTC	XMR	XRP	NMC	FTC	PPC	FROM
BTC	47.83	7.04	2.74	4.17	3.26	3.86	3.98	3.12	52.17	BTC	18.06	15.82	2.89	5.32	3.61	17.79	8.03	2.28	81.94
ETH	19.65	18.23	5.03	5.42	3.72	3.74	5.02	5.00	81.77	ETH	10.05	19.34	4.19	5.30	4.06	19.08	9.57	2.51	80.66
LTC	21.04	9.68	11.88	11.36	4.62	5.32	4.44	3.76	88.12	LTC	10.71	14.26	6.14	6.96	3.67	25.39	11.46	2.06	93.86
XMR	33.15	5.48	2.25	18.14	4.18	3.61	3.66	4.90	81.86	XMR	15.97	15.34	3.18	13.59	3.95	15.26	8.08	2.19	86.41
XRP	4.89	6.94	3.19	7.84	10.40	4.06	5.56	5.11	89.60	XRP	7.82	16.64	3.91	5.68	5.43	20.78	12.01	2.00	94.57
NMC	4.02	5.18	7.64	9.72	5.15	13.89	5.59	5.32	86.11	NMC	7.24	15.86	4.59	5.47	4.25	24.28	13.25	2.06	75.72
FTC	6.85	4.70	4.02	9.54	4.65	3.60	7.52	5.70	92.48	FTC	8.49	20.72	3.86	5.78	4.91	14.49	11.32	2.15	88.68
PPC	6.06	4.60	2.81	9.70	5.96	3.65	5.29	6.33	93.67	PPC	7.54	18.11	3.37	8.15	3.83	15.78	11.71	2.69	97.31
TO	100.97	48.25	31.38	66.99	36.94	31.07	38.23	37.58	73.97	TO	76.26	137.99	29.67	48.44	32.76	142.31	84.12	17.26	77.68

Note: Table 4 presents the forecast error variance decomposition (FEVD) results under low political uncertainty periods (left panel) and high political uncertainty periods (right panel). Off-diagonal elements represent cross-cryptocurrency spillovers. The “FROM” row indicates the total spillover received by a cryptocurrency from all others. The “TO” column indicates the total spillover transmitted by a cryptocurrency to all others. The bottom-right cell shows the total spillover index. All values are expressed in percentages. (Tables 5–8 are similar.)

However, in the short term (D6-D8), Although this is still the case on the whole, individual case is different. The risk spillover effect under the scale of D7 is 76.76% in the periods of low political uncertainty and 75.89% in the periods of high uncertainty. This short-term and long-term difference may reflect the short-term behavior adjustment of investors in the face of high uncertainty, resulting in temporary changes in market volatility patterns in the short term, while the long-term enhancement effect reveals the persistent impact of uncertainty on the relevance of the cryptocurrency market, which should be the product of investors deepening or adjusting their perception of risk.

The “TO” row of the matrix explains how much one cryptocurrency affects the volatility of other cryptocurrencies., while “FROM” explains the degree to which the volatility of one cryptocurrency is affected by other cryptocurrencies. The results partially confirm but also partially revise the hypothesis H3. (Large cryptocurrencies are stable risk transmitters, while small cryptocurrencies are more vulnerable risk receivers)

Long-term perspective (D1, D2 scale): The role of cryptocurrencies is plastic. In a period of low political uncertainty, BTC (D1: 141.31%) is the core of risk spillover, followed by ETH (D1: 73.28%). Interestingly, in high political uncertainty periods, the contribution of BTC to the volatility of other cryptocurrencies declines significantly, with the D1 scale down to 92.79% and the D2 scale only 30.85%.

Mid-term (D3-D5 scale): We discover that BTC are always the main contributor in both political uncertainty periods of political uncertainty. XMR and ETH were also relatively large contributors, and they are relatively less affected by other cryptocurrencies.

Short term (D6-D8 scale): information indicates that the center of risk spillovers is variable. For example, under the condition of high political uncertainty, NMC unexpectedly becomes the largest contributor (142.31%) in the D8 level. This indicates that short-term market conditions may be greatly affected by market sentiment or specific events.

In addition, in two periods of political uncertainty, PPC and NMC are usually vulnerable to the impact of other cryptocurrencies, but their contribution to the volatility of other cryptocurrencies is relatively small. This clearly confirms the view in hypothesis H3 that small cryptocurrencies are more likely to become vulnerable risk recipients. Although the view that large cryptocurrencies are stable risk transmitters is basically correct (such as BTC and ETH), XMR is not a large cryptocurrency, and its risk transmission ability is also strong, which may be related to it being a privacy coin.

Our findings complement previous research: Qiao *et al*. study how cryptocurrencies are interrelated and find that cryptocurrencies with a long history and high market recognition such as Bitcoin are more likely to affect the development of other cryptocurrencies [16]. Our study complements this. We find that in the context of political uncertainty, BTC is a major risk contributor to other cryptocurrencies during periods of low political uncertainty, but its risk leadership declines significantly during periods of high political uncertainty.

### 5.3 Risk spillover network

– plot the multiscale risk spillover networks in different political uncertainty periods. The size of the nodes represents the magnitude of the risk spillover strength. The arrows indicate the direction of the risk spillover (sender → receiver), and the thickness of the lines between the nodes is used to interpret the strength of the risk spillover between the variables. The results show that political uncertainty has a significant impact on the risk spillover network of the cryptocurrency market, and will change the leader and network structure of risk spillover, indicating that risk transmission is asymmetric.

**Fig 5:**
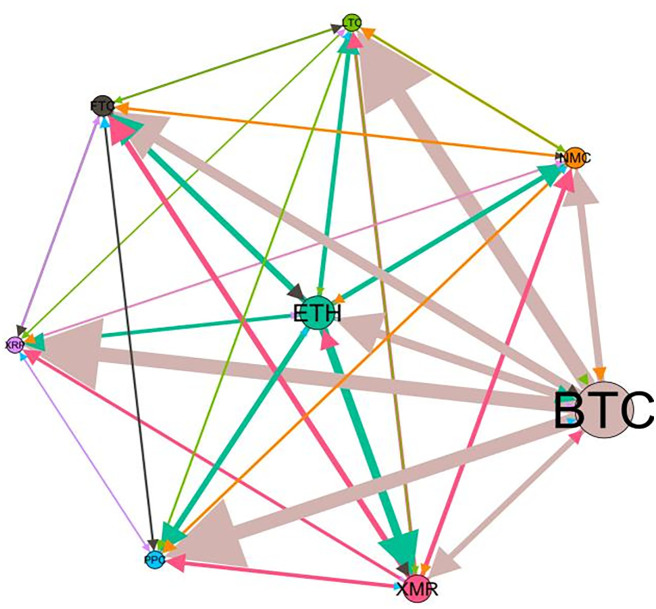
Network at D_1_ (low).

**Fig 6:**
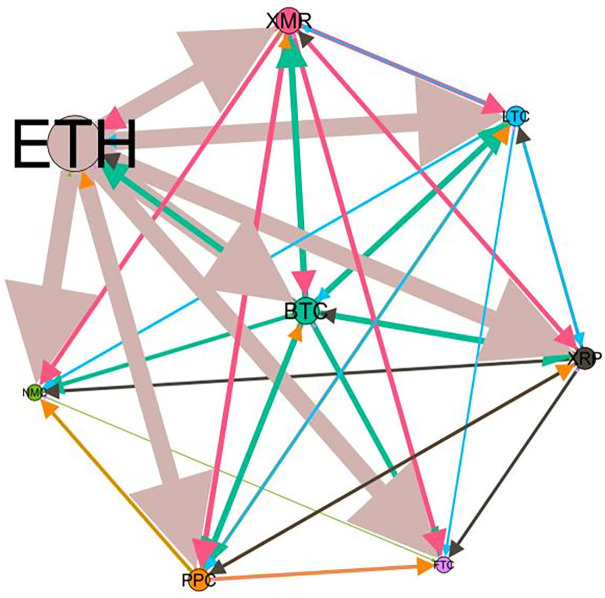
Network at D_1_ (high).

**Fig 7:**
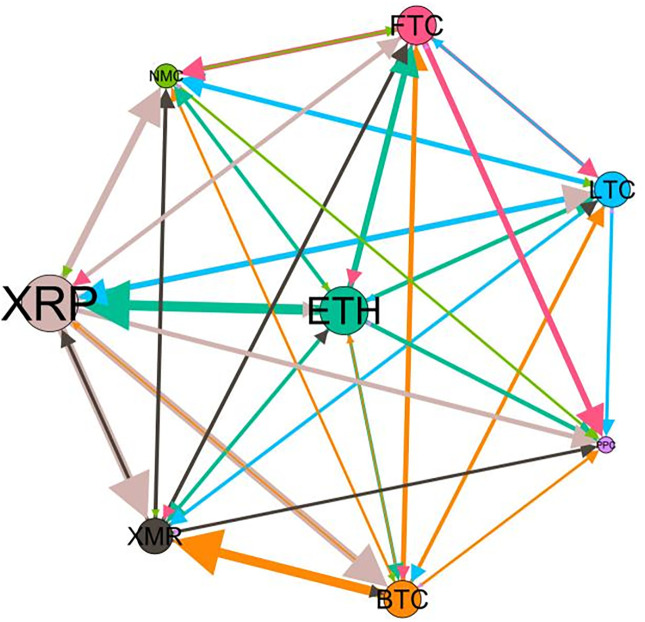
Network at D_2_ (low).

**Fig 8:**
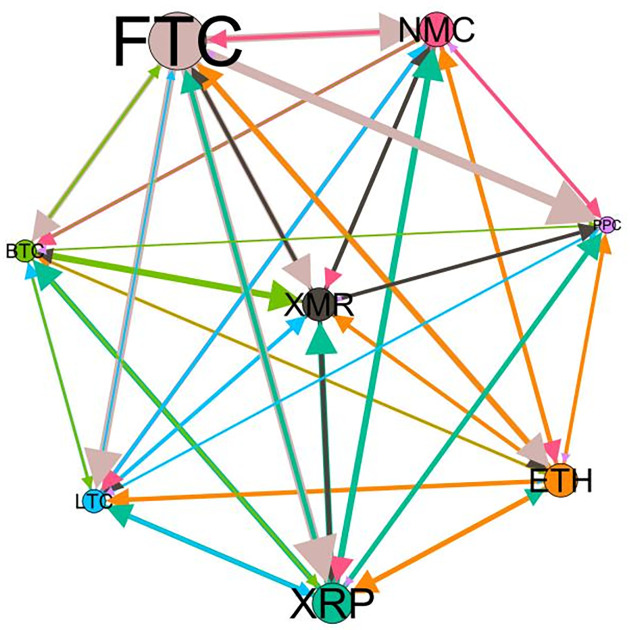
Network at D_2_ (high).

**Fig 9:**
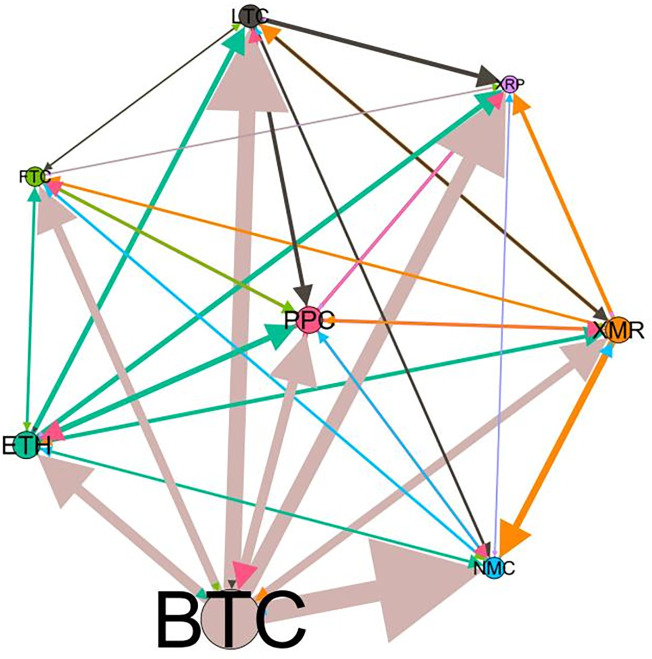
Network at D_3_ (low).

**Fig 10:**
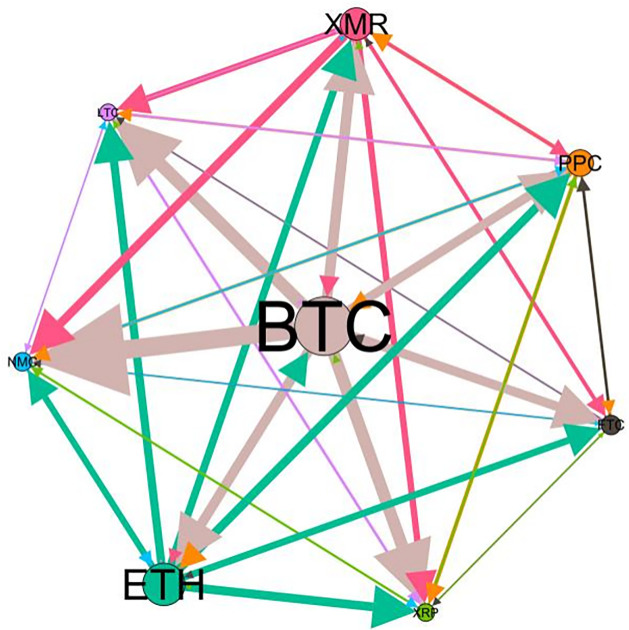
Network at D_3_ (high).

**Fig 11:**
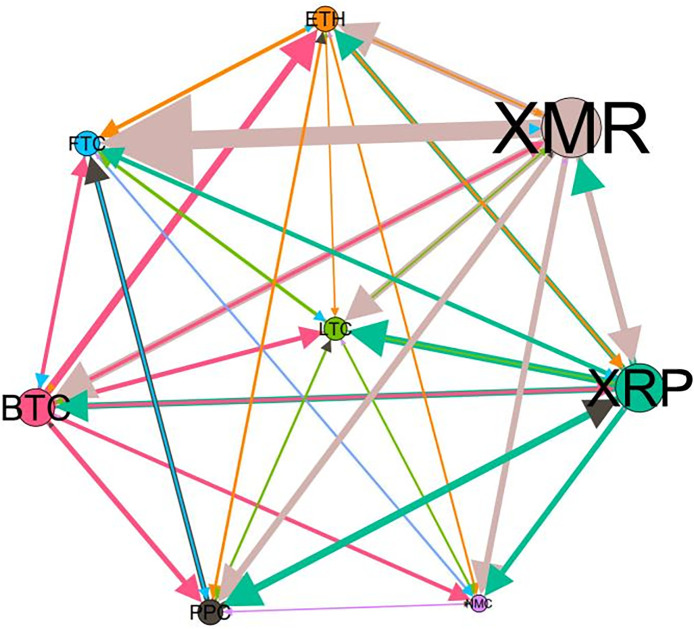
Network at D_4_ (low).

**Fig 12:**
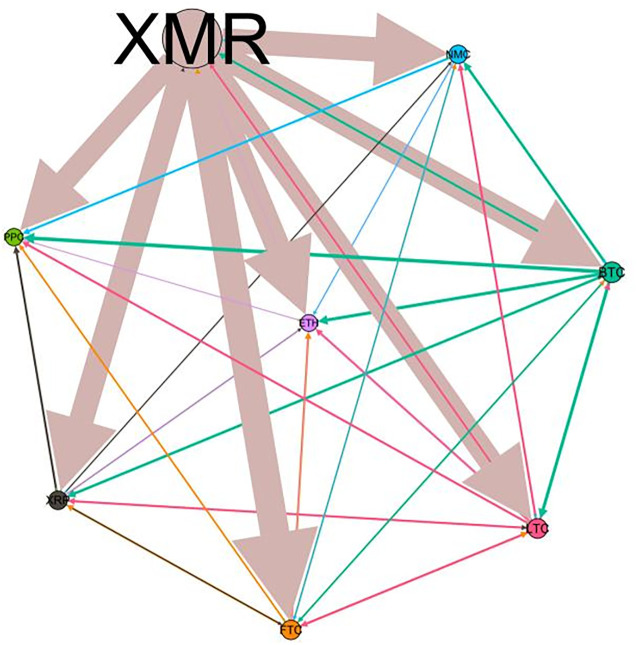
Network at D_4._

**Fig 13:**
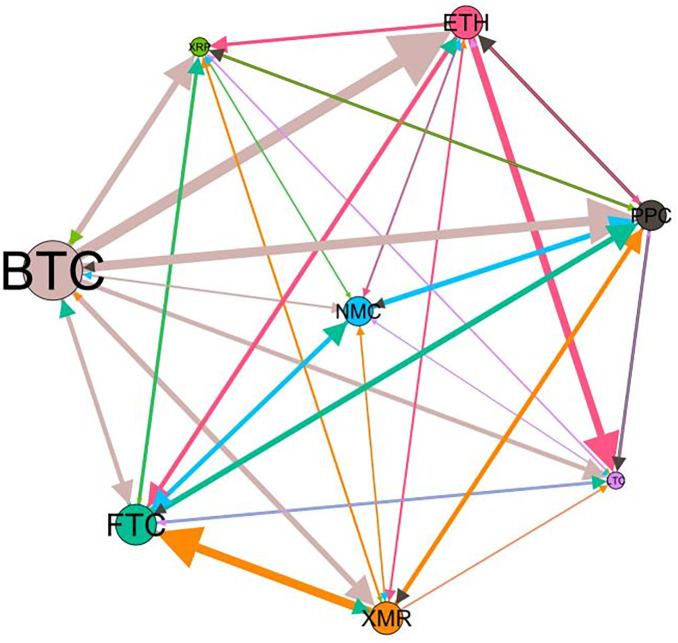
Network at D_5_ (low).

**Fig 14:**
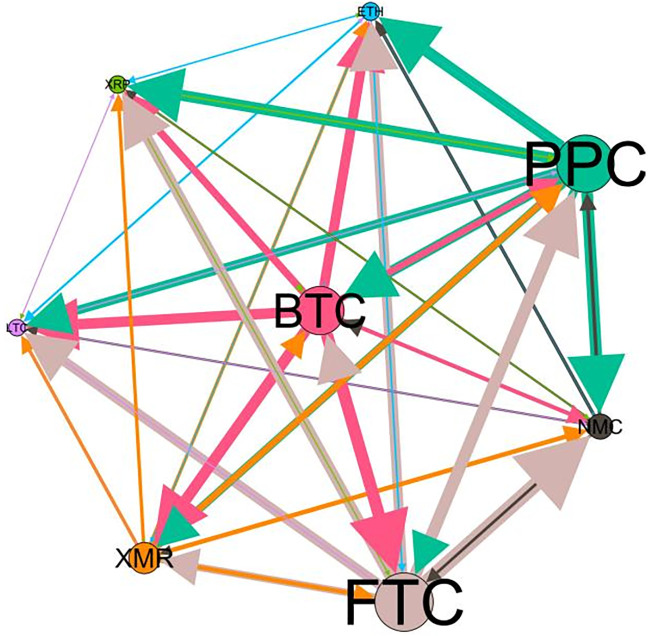
Network at D_5_ (high).

**Fig 15:**
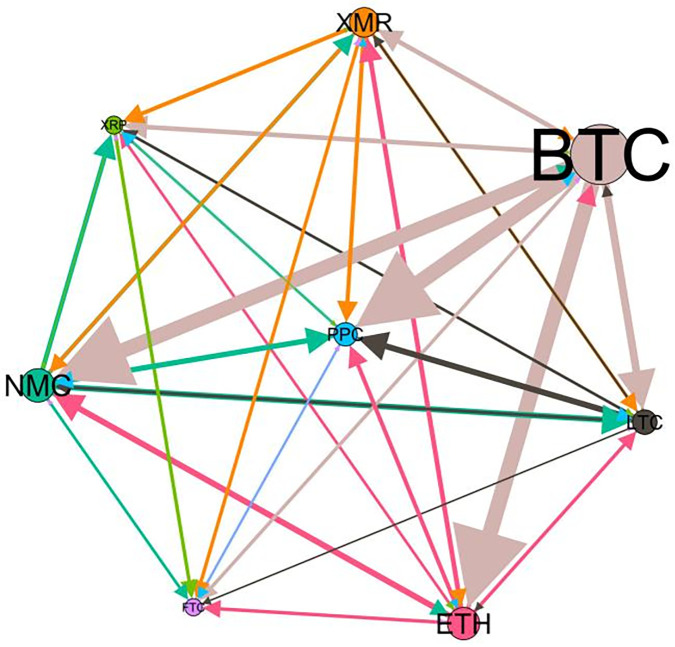
Network at D_6_ (low).

**Fig 16:**
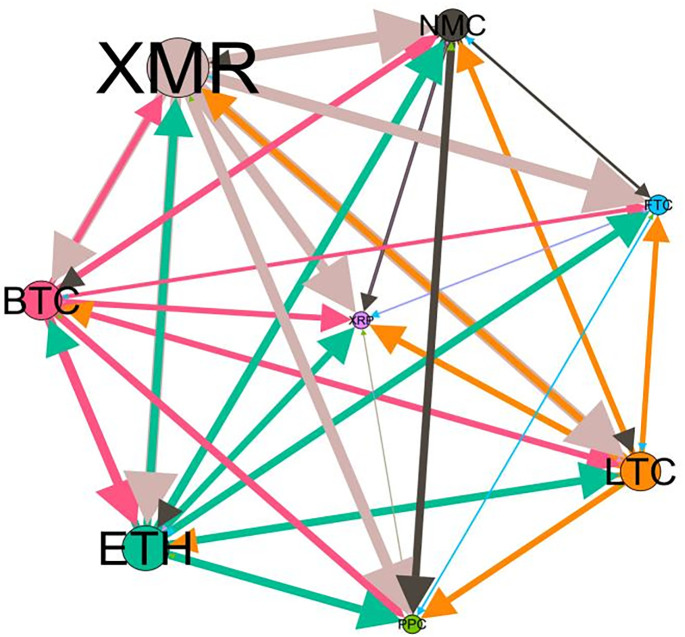
Network at D_6_ (high).

**Fig 17:**
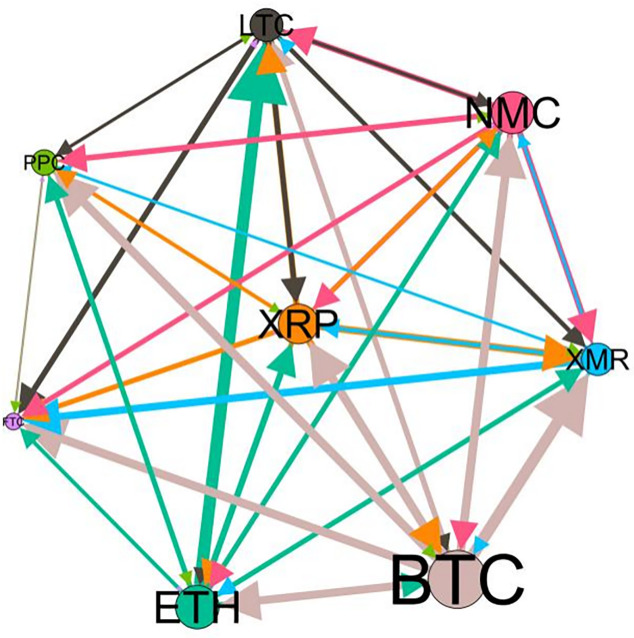
Network at D_7_ (low).

**Fig 18:**
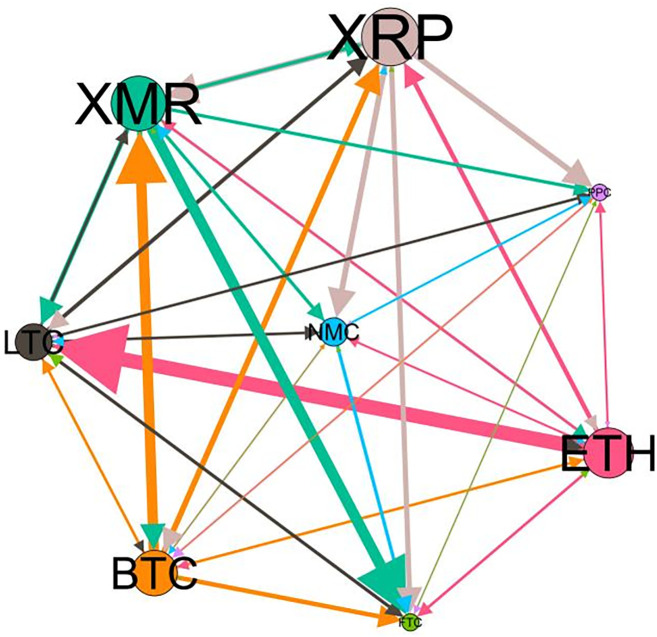
Network at D_7_ (high).

**Fig 19:**
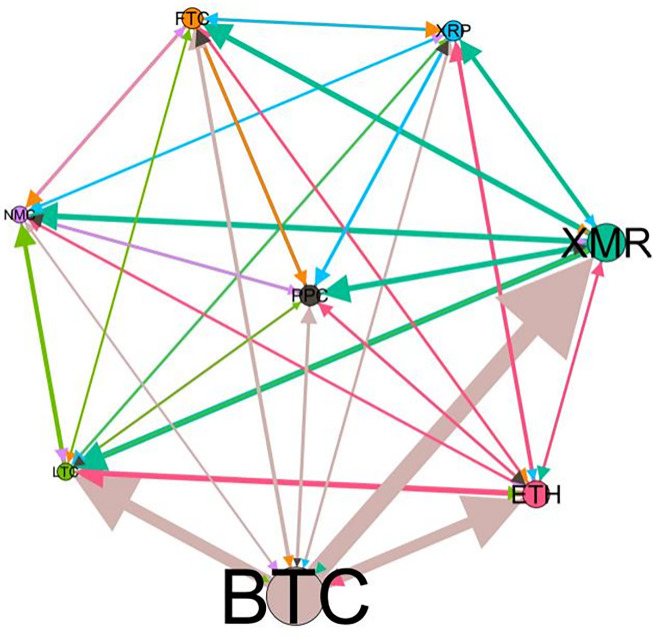
Network at D_8_ (low).

**Fig 20:**
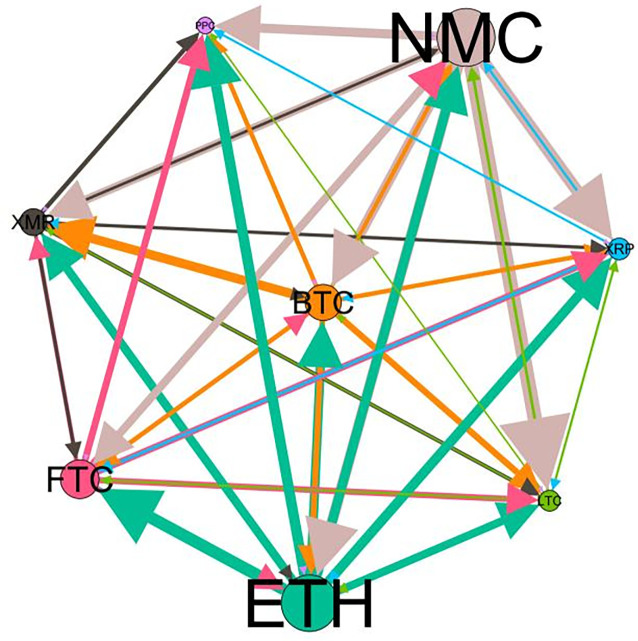
Network at D_8_ (high). Note: Figs. 5–20 visualize the risk spillover network of eight cryptocurrencies in D1-D8 (different time scales) during low or high political uncertainty. The pictures on the left represent low political uncertainty and the right represents high political uncertainty. The node size represents the size of the total risk spillover intensity. The arrow direction represents the direction of the risk spillover (sender to receiver). The thickness of the line represents the intensity of the two overflows, and the thicker the line indicates the stronger the overflow.

On the long-term scale, BTC, ETH and XRP are more influential risk transmitters, and These three cryptocurrencies generally exhibit significant spillover effects between each pair. In D1, BTC is the main contributor to risk spillover in the periods of low political uncertainty, and ETH becomes the risk spillover center in the periods of high uncertainty. Comparing the above findings with previous studies, Moratis (2020) finds that BTC dominates the risk spillover of cryptocurrencies, but scale is not the only determinant of the impact. This study complements and corrects this. Under the impact of political uncertainty, not only BTC, ETH and XMR also show stable risk transmission in the cryptocurrency market. The leading role of various cryptocurrencies in risk transmission changes dynamically with political uncertainty.

In the short-term and medium-term, BTC is the main risk exporter in two periods of political uncertainty, with a strong risk association with ETH and XMR, while ETH and XMR are also important risk spillers that cannot be ignored. NMC and PPC are greatly impacted by other cryptocurrencies. Its economic significance is that NMC is vulnerable and easy to become a risk absorption pool, and regulatory attention should be paid to its potential to trigger greater systemic risks.

The results show that political uncertainty has a significant impact on the risk spillovers network of the cryptocurrency market. The role of cryptocurrencies can change according to political uncertainty conditions. In general, BTC, ETH and XMR show stable risk transmission in the cryptocurrency market, while PPC and NMC are more vulnerable risk receivers. Therefore, when faced with a changing political environment, investors should adjust their portfolio preferences according to the time domain. This further proves that the hypothesis H3 is partially true. Small cryptocurrencies are more likely to become vulnerable risk recipients. Although the view that large cryptocurrency is a stable risk transmitter is basically correct (such as BTC and ETH), XMR is not a large cryptocurrency and also has strong risk transmission ability. This should be related to the fact that XMR is a privacy cryptocurrency and has received considerable attention.

### 5.4 Robustness checks

A robustness check of the main results is conducted. First, we modify the number of lags in the STVAR. The original number of lags is 2; for the robustness check, we change it to 1 and 3. The results of the partial FEVD for this test are shown in Tables 5 and 6. Additionally, we adjust the horizon for impulse responses. The original horizon for impulse responses is 15; for the robustness check, we set it to 13 and 17. The results of the partial FEVD for this test are shown in Tables 7 and 8.

**Table 5:** Risk spillover effect matrix of cryptocurrency in different policy uncertainty periods (number of lags = 1).

In low political uncertainty periods										In high political uncertainty periods									
Scale D_8_	BTC	ETH	LTC	XMR	XRP	NMC	FTC	PPC	FROM	Scale D_8_	BTC	ETH	LTC	XMR	XRP	NMC	FTC	PPC	FROM
BTC	53.33	4.91	1.51	1.30	9.59	5.19	0.60	3.45	46.67	BTC	21.97	5.00	1.79	2.34	6.24	29.03	1.18	1.81	78.03
ETH	18.15	26.47	1.89	1.82	13.00	5.80	1.10	4.03	73.53	ETH	8.94	12.04	2.30	3.71	6.00	28.76	2.64	2.07	87.96
LTC	19.60	7.08	28.02	3.56	7.46	7.04	1.07	4.47	71.98	LTC	14.57	6.81	13.50	2.13	3.71	34.16	1.40	1.36	86.50
XMR	31.88	7.06	1.74	20.04	10.02	5.23	0.78	5.54	79.96	XMR	19.12	5.36	1.91	15.59	5.44	21.72	1.89	1.95	84.41
XRP	5.51	11.74	1.82	1.53	29.20	3.88	1.19	4.10	70.80	XRP	2.49	8.17	2.41	2.61	11.94	24.42	2.92	1.68	88.06
NMC	3.02	9.41	4.62	3.33	18.00	18.14	1.11	4.72	81.86	NMC	2.51	4.99	1.68	1.57	5.62	43.73	1.33	1.99	56.27
FTC	6.05	6.09	1.33	2.36	18.20	5.50	5.38	5.88	94.62	FTC	2.75	5.05	1.29	1.64	5.71	32.01	6.04	2.13	93.96
PPC	6.66	6.32	1.64	2.22	22.74	6.77	1.13	7.13	92.87	PPC	2.78	4.05	1.04	1.29	7.84	36.18	1.26	2.97	97.03
TO	96.13	59.94	15.64	18.65	120.43	45.37	8.02	36.67	68.03	TO	55.72	44.54	13.33	16.79	47.01	240.53	14.30	14.99	74.69

**Table 6:** Risk spillovers effect matrix of cryptocurrency in different policy uncertainty periods (number of lags = 3).

In low political uncertainty periods										In high political uncertainty periods									
Scale D_2_	BTC	ETH	LTC	XMR	XRP	NMC	FTC	PPC	FROM	Scale D_2_	BTC	ETH	LTC	XMR	XRP	NMC	FTC	PPC	FROM
BTC	18.80	8.35	6.75	6.06	9.92	4.82	5.02	3.36	81.20	BTC	12.66	9.99	7.92	10.13	8.42	6.67	9.58	2.81	87.34
ETH	4.63	21.18	4.98	5.31	5.69	4.35	5.45	3.20	78.82	ETH	2.03	17.12	7.33	9.61	6.54	7.24	10.14	2.27	82.88
LTC	6.04	11.71	14.30	6.37	8.20	4.81	5.33	3.04	85.70	LTC	3.29	15.14	12.69	10.14	6.63	7.46	9.70	2.44	87.31
XMR	11.39	10.51	6.19	11.73	9.54	4.32	5.00	3.23	88.27	XMR	7.64	12.65	8.19	12.93	8.33	6.31	9.05	2.48	87.07
XRP	4.82	17.46	7.40	5.91	12.40	4.35	5.18	2.84	87.60	XRP	2.61	17.44	8.45	9.88	8.80	7.85	10.00	2.52	91.20
NMC	5.28	9.05	9.73	7.40	9.58	10.02	6.32	3.36	89.98	NMC	2.36	9.54	9.93	12.12	10.01	8.95	11.09	3.25	91.05
FTC	7.65	10.04	5.23	7.08	6.18	5.29	11.22	3.39	88.78	FTC	3.83	11.92	7.03	10.07	6.61	7.99	13.37	2.39	86.63
PPC	5.32	8.13	6.98	6.18	6.69	6.12	9.83	8.30	91.70	PPC	3.05	10.23	7.63	10.60	6.42	8.54	12.11	6.88	93.12
TO	49.39	81.61	52.09	50.29	61.39	38.53	46.69	25.42	76.89	TO	26.27	96.06	63.61	84.52	58.86	58.66	81.27	20.84	78.51

**Table 7:** Risk spillovers effect matrix of cryptocurrency in different policy uncertainty periods (Horizon for impulse responses = 13).

In low political uncertainty periods										In high political uncertainty periods									
Scale D_3_	BTC	ETH	LTC	XMR	XRP	NMC	FTC	PPC	FROM	_Scale D_3__	BTC	ETH	LTC	XMR	XRP	NMC	FTC	PPC	FROM
BTC	55.32	4.36	2.93	4.28	1.38	2.13	3.54	7.57	44.68	BTC	45.52	9.13	1.41	5.18	1.95	0.94	3.18	4.99	54.48
ETH	16.35	35.57	2.01	3.52	1.64	3.93	2.85	6.10	64.43	ETH	13.36	35.37	0.74	3.41	1.65	4.37	2.10	6.75	64.63
LTC	27.82	8.47	9.97	6.48	2.00	3.08	3.19	4.32	90.03	LTC	19.13	11.32	6.37	8.83	2.22	2.68	2.59	4.03	93.63
XMR	14.49	7.02	5.23	13.05	2.38	4.54	1.65	5.03	86.95	XMR	14.53	10.52	2.10	16.98	2.23	1.85	2.74	5.16	83.02
XRP	26.76	9.20	8.39	7.02	6.36	3.17	2.88	5.44	93.64	XRP	18.59	12.29	4.86	8.54	6.10	2.96	2.45	5.47	93.90
NMC	37.69	4.22	4.48	11.63	2.53	13.07	3.44	4.27	86.93	NMC	29.45	9.29	3.07	11.92	3.40	10.77	2.32	4.00	89.23
FTC	14.26	5.00	2.94	4.57	2.03	4.49	5.99	3.97	94.01	FTC	12.83	10.13	1.00	5.32	1.33	1.77	10.15	3.58	89.85
PPC	15.73	9.95	7.14	3.86	2.48	3.49	4.50	25.89	74.11	PPC	13.08	12.74	4.37	4.85	3.46	3.31	3.96	15.00	85.00
TO	166.51	53.05	35.73	45.62	16.25	29.99	23.66	41.39	70.53	TO	132.36	85.13	18.49	53.90	17.26	20.00	23.23	39.15	72.64
**Scale D**_**6**_	BTC	ETH	LTC	XMR	XRP	NMC	FTC	PPC	FROM	**Scale D_6_**	BTC	ETH	LTC	XMR	XRP	NMC	FTC	PPC	FROM
BTC	47.76	7.67	3.35	4.39	2.31	4.55	2.26	5.33	52.24	BTC	24.79	11.46	10.61	15.11	1.27	8.20	2.78	2.15	75.21
ETH	28.45	15.34	2.86	5.65	3.19	5.16	3.49	2.83	84.66	ETH	17.32	15.51	8.76	18.48	1.41	8.89	4.03	2.40	84.49
LTC	14.62	6.47	6.15	4.97	2.68	8.90	1.96	4.80	93.85	LTC	10.82	12.66	11.75	18.72	1.43	8.42	3.83	2.10	88.25
XMR	9.13	8.74	3.38	16.49	2.42	5.72	3.51	2.74	83.51	XMR	10.34	13.20	10.85	23.06	1.25	7.17	4.12	2.00	76.94
XRP	8.60	5.28	4.67	6.28	17.84	6.80	2.29	4.38	82.16	XRP	9.46	11.90	10.94	18.21	6.94	6.16	3.84	2.09	93.06
NMC	26.43	10.13	5.29	5.63	1.96	17.70	2.01	6.16	82.30	NMC	12.20	14.73	10.05	19.03	1.34	16.41	3.78	2.94	83.59
FTC	5.83	6.02	2.97	5.85	5.83	3.46	34.51	3.71	65.49	FTC	6.83	13.40	8.79	20.69	2.43	4.92	16.33	2.10	83.67
PPC	28.26	7.79	10.50	6.34	2.18	7.85	2.21	9.99	90.01	PPC	13.92	14.05	10.97	17.82	1.18	13.73	4.22	4.57	95.43
TO	123.23	57.63	36.68	44.61	22.71	49.85	21.49	33.31	70.47	TO	84.63	103.42	82.67	147.18	11.49	64.61	30.97	17.48	75.63

**Table 8:** Risk spillovers effect matrix of cryptocurrency in different policy uncertainty periods (Horizon for impulse responses = 17).

In low political uncertainty periods										In high political uncertainty periods									
Scale D_1_	BTC	ETH	LTC	XMR	XRP	NMC	FTC	PPC	FROM	Scale D_1_	BTC	ETH	LTC	XMR	XRP	NMC	FTC	PPC	FROM
BTC	33.49	6.06	3.73	5.26	2.82	6.81	4.36	3.83	66.51	BTC	14.98	35.61	5.35	9.96	7.36	1.91	1.69	7.49	85.02
ETH	17.11	16.79	3.52	7.31	2.46	5.06	6.78	3.89	83.21	ETH	13.04	36.79	5.29	10.09	7.68	1.78	2.03	7.16	63.21
LTC	27.63	6.53	4.47	6.25	2.25	6.15	4.58	3.81	95.53	LTC	13.12	35.87	5.88	10.03	7.63	1.88	1.69	7.65	94.12
XMR	12.09	13.85	2.88	13.68	1.95	5.39	5.50	3.73	86.32	XMR	13.10	36.45	3.86	12.49	6.52	1.77	1.85	5.70	87.51
XRP	27.13	6.23	2.64	6.43	5.62	5.30	4.59	4.07	94.38	XRP	12.75	35.58	5.40	9.81	9.22	1.79	1.72	7.51	90.78
NMC	13.83	7.30	2.95	7.83	2.18	14.25	3.40	4.01	85.75	NMC	8.59	38.57	5.42	9.34	7.70	3.84	1.64	8.03	96.16
FTC	18.79	9.65	3.09	9.60	2.46	6.70	6.92	4.28	93.08	FTC	12.19	35.76	4.94	10.34	7.36	1.94	2.83	7.67	97.17
PPC	27.10	8.71	4.31	6.91	2.03	5.94	4.46	9.76	90.24	PPC	13.51	34.92	5.67	11.62	7.24	1.88	1.72	7.91	92.09
TO	149.91	63.97	25.66	54.79	17.99	47.54	37.66	31.95	77.22	TO	92.55	288.09	40.35	82.92	58.00	14.94	14.08	57.55	78.45
**Scale D**_**5**_	BTC	ETH	LTC	XMR	XRP	NMC	FTC	PPC	FROM	**Scale D_5_**	BTC	ETH	LTC	XMR	XRP	NMC	FTC	PPC	FROM
BTC	27.91	2.21	1.49	2.70	4.88	3.05	5.13	3.46	72.09	BTC	18.78	0.97	1.29	8.06	2.66	6.85	17.21	20.04	81.22
ETH	18.68	12.86	3.06	3.25	2.53	4.30	5.48	5.58	87.14	ETH	15.22	6.25	1.62	5.75	1.63	6.07	18.71	22.36	93.75
LTC	7.75	11.76	14.51	2.88	2.69	2.73	4.83	4.42	85.49	LTC	17.43	4.38	5.91	5.72	1.51	4.40	16.71	16.76	94.09
XMR	9.97	3.66	1.58	20.14	2.12	3.29	5.92	4.57	79.86	XMR	19.46	2.04	1.18	10.46	1.36	4.58	14.03	15.29	89.54
XRP	10.92	5.60	2.80	3.96	17.01	3.27	5.35	4.83	82.99	XRP	12.31	2.82	1.83	5.65	6.14	4.75	19.41	21.21	93.86
NMC	4.39	3.14	1.71	3.18	2.01	16.73	6.45	3.72	83.27	NMC	5.85	1.15	1.78	6.74	1.21	11.02	24.46	21.61	88.98
FTC	7.55	7.02	2.97	13.20	2.57	6.85	18.38	3.88	81.62	FTC	21.44	2.90	1.81	7.76	1.74	5.53	16.54	14.85	83.46
PPC	14.66	2.52	2.06	7.07	3.44	8.39	8.80	16.14	83.86	PPC	10.26	0.95	1.84	9.63	2.01	6.86	21.60	26.29	73.71
TO	79.59	38.86	17.65	38.99	22.78	35.93	46.82	34.93	72.92	TO	107.64	16.49	12.64	54.52	13.43	43.92	151.51	155.41	77.62

The results reveal that political uncertainty shocks have an asymmetric effect on cryptocurrencies. In most cases, the risk spillovers in the cryptocurrency market can be increased by a shock during high political uncertainty periods. Furthermore, BTC, ETH, and XMR are stable risk transmitters while NMC and PPC are more vulnerable risk receivers. This result is consistent with those shown in Table 3.

## 6. Conclusion

This study relies on the STVAR model to capture the impact of political uncertainty on the cryptocurrency market. It also employs the network topology and wavelet packet-based approaches to capture the multiscale risk spillovers in the cryptocurrency market. The results mainly reveal the following: (1) The reaction of cryptocurrencies to the impact of political uncertainty is significantly asymmetric. As the identified medium and long-term components show, most cryptocurrencies react more strongly in periods of high political uncertainty than in periods of low uncertainty, which also shows that the impact of political uncertainty on cryptocurrencies is long-term and persistent, and poses a certain challenge to holding cryptocurrencies as long-term safe haven assets. (2) The shocks in the period of high political uncertainty expand the risk spillover across cryptocurrencies. This shows that high political uncertainty has increased the systemic risk level of the entire cryptocurrency market, and also shows that the external environment will affect the risk transmission within the cryptocurrency. (3) BTC, ETH and XMR are stable risk transmitters, while PPC and NMC are more vulnerable risk receivers. When faced with changing political uncertainty, BTC is not an immutable leader, and investors should adjust their portfolio preferences. Risk-averse investors should choose a more stable cryptocurrency type for investment.

These insights are of great significance to cryptocurrency investors and regulatory decision makers. Firstly, for investors: Based on finding (1), that cryptocurrencies exhibit significant asymmetry in their response to political uncertainty shocks, investors should incorporate political uncertainty into their asset allocation models, pay close attention to political events, and long-term investors may appropriately reduce their overall position when facing high political uncertainty, rather than blindly increasing their positions. Based on finding (2), that shocks during periods of high political uncertainty amplify risk spillovers among cryptocurrencies, investors can choose to invest in assets with lower correlation to cryptocurrencies during periods of high political uncertainty to reduce risk. Based on finding (3), that BTC, ETH, and XMR are stable risk propagators, while PPC and NMC are more likely to become risk recipients, investors can improve their asset allocation by investing primarily in BTC, ETH, and XMR as the main cryptocurrencies to maintain the stability of returns.

However, this study also has limitations and needs further improvement. Firstly, although this article analyzes the impact of political uncertainty on risk spillover in the cryptocurrency market, it does not delve into its impact mechanism and channels. Secondly, the sample in this article is from 2015 to 2024. In the future, with the development of the cryptocurrency market, an increase in sample size may have a certain impact on the research results. Additionally, the model may not be able to capture all the impacts of political events. Future research could expand on data sources and methodologies by, for example, incorporating broader variables related to political or economic uncertainty to improve the predictive accuracy and applicability of the model, or adopting more precise methods, can also focus on characterizing sudden political events.

## References

[1] Chen C, Liu L, Zhao N. Fear sentiment, uncertainty, and bitcoin price dynamics: the case of COVID-19. Research on pandemics. Routledge; 2021. 166–77.

[2] Zhang X, Ding Z. Multiscale systemic risk and its spillover effects in the cryptocurrency market. Complexity. 2021;2021(1). doi: 10.1155/2021/5581843

[3] Dagher L, Rao A, Vishal D, Shobande O. Geopolitics, uncertainty, and cryptocurrency: a love triangle gone wrong. Appl Finan Lett. 2024;13:48–62. doi: 10.24135/afl.v13i.699

[4] Muhammad U, Chi-Wei S, Abbas RSK, Xue-Feng S. Bitcoin: a safe haven asset and a winner amid political and economic uncertainties in the US?. Tech Forecast Soc Change. 2021;167:120680.

[5] He C, Li Y, Wang T, Shah SA. Is cryptocurrency a hedging tool during economic policy uncertainty? An empirical investigation. Humanit Soc Sci Commun. 2024;11(1). doi: 10.1057/s41599-023-02532-x

[6] Sharma AK. Asymmetric impact of economic policy uncertainty on cryptocurrency market: Evidence from NARDL approach. J Econ Asymmetries. 2023;27:e00298. doi: 10.1016/j.jeca.2023.e00298

[7] Kilci EN, Yilanci V. Do uncertainties and risks have an impact on cryptocurrency returns? Evidence from the symmetric and asymmetric fourier quantile causality test. Estudios de Economía. 2025;52(1):27–58. doi: 10.4067/s0718-52862025000100027

[8] Borri N. Conditional tail-risk in cryptocurrency markets. J Emp Finance. 2019;50:1–19. doi: 10.1016/j.jempfin.2018.11.002

[9] Katsiampa P. An empirical investigation of volatility dynamics in the cryptocurrency market. Res Inter Business Finance. 2019;50:322–35. doi: 10.1016/j.ribaf.2019.06.004

[10] Luu Duc Huynh T. Spillover risks on cryptocurrency markets: a look from VAR-SVAR granger causality and student’s-t copulas. J Risk Finan Manag. 2019;12(2):52. doi: 10.3390/jrfm12020052

[11] Günther S, Fieberg C, Poddig T. The cross-section of cryptocurrency risk and return. Vierteljahrshe zur Wirtschaft. 2020;89(4):7–28. doi: 10.3790/vjh.89.4.7

[12] Xu Q, Zhang Y, Zhang Z. Tail-risk spillovers in cryptocurrency markets. Finance Res Letters. 2021;38:101453. doi: 10.1016/j.frl.2020.101453

[13] Gençay R, Gradojevic N, Selçuk∥ F, Whitcher B. Asymmetry of information flow between volatilities across time scales. Quant Finance. 2010;10(8):895–915. doi: 10.1080/14697680903460143

[14] Fruehwirt W, Hochfilzer L, Weydemann L, Roberts S. Cumulation, crash, coherency: a cryptocurrency bubble wavelet analysis. Finance Res Letters. 2020;:101668.

[15] Omane-Adjepong M, Alagidede P, Akosah NK. Wavelet time-scale persistence analysis of cryptocurrency market returns and volatility. Physica A: Stat Mech Appl. 2019;514:105–20. doi: 10.1016/j.physa.2018.09.013

[16] Qiao X, Zhu H, Hau L. Time-frequency co-movement of cryptocurrency return and volatility: Evidence from wavelet coherence analysis. Int Rev Finan Anal. 2020;71:101541. doi: 10.1016/j.irfa.2020.101541

[17] Balcilar M, Bouri E, Gupta R, Roubaud D. Can volume predict Bitcoin returns and volatility? A quantiles-based approach. Economic Model. 2017;64:74–81. doi: 10.1016/j.econmod.2017.03.019

[18] Cheng CHJ, Hankins WB, Chiu C-W. Does US partisan conflict matter for the Euro area?. Economics Letters. 2016;138:64–7. doi: 10.1016/j.econlet.2015.11.030

[19] Gupta R, Pierdzioch C, Selmi R, Wohar ME. Does partisan conflict predict a reduction in US stock market (realized) volatility? Evidence from a quantile-on-quantile regression model☆. The North American J Econ Finance. 2018;43:87–96. doi: 10.1016/j.najef.2017.10.006

[20] Ankam PK, Pattanayak JK. Do cryptocurrencies exhibit similar hedging and safe haven properties under different uncertainty indices? A comparative analysis with gold and the U.S. Dollar index. Finance Res Letters. 2025;86:108934. doi: 10.1016/j.frl.2025.108934

[21] de Almeida IN, Palazzi RB, Klotzle MC. Breaking from the herd: evidence from the 2024 U.S. election. Economics Letters. 2026;259:112789. doi: 10.1016/j.econlet.2025.112789

[22] Azzimonti M. Partisan conflict and private investment. J Monetary Econ. 2018;93:114–31. doi: 10.1016/j.jmoneco.2017.10.007

[23] Diebold FX, YiLmaz K. On the network topology of variance decompositions: measuring the connectedness of financial firms. J Econom. 2014;182:119–34.

[24] Ji Q, Bouri E, Lau CKM, Roubaud D. Dynamic connectedness and integration in cryptocurrency markets. Inter Rev Finan Anal. 2019;63:257–72. doi: 10.1016/j.irfa.2018.12.002

[25] Koutmos D. Return and volatility spillovers among cryptocurrencies. Econ Letters. 2018;173:122–7. doi: 10.1016/j.econlet.2018.10.004

[26] Moratis G. Quantifying the spillover effect in the cryptocurrency market. Finance Res Letters. 2021;38:101534. doi: 10.1016/j.frl.2020.101534

[27] Yi S, Xu Z, Wang G-J. Volatility connectedness in the cryptocurrency market: is Bitcoin a dominant cryptocurrency?. Inter Rev Financial Anal. 2018;60:98–114. doi: 10.1016/j.irfa.2018.08.012

[28] Foglia M, Dai P-F. “Ubiquitous uncertainties”: spillovers across economic policy uncertainty and cryptocurrency uncertainty indices. J Asian Business Econom Stud. 2021;29(1):35–49. doi: 10.1108/jabes-05-2021-0051

[29] Colon F, Kim C, Kim H, Kim W. The effect of political and economic uncertainty on the cryptocurrency market. Finance Res Letters. 2021;39:101621. doi: 10.1016/j.frl.2020.101621

[30] Wu W, Tiwari AK, Gozgor G, Leping H. Does economic policy uncertainty affect cryptocurrency markets? Evidence from Twitter-based uncertainty measures. Res Inter Business Finance. 2021;58:101478. doi: 10.1016/j.ribaf.2021.101478

[31] Zhang X, Yang X, He Q. Multi-scale systemic risk and spillover networks of commodity markets in the bullish and bearish regimes. North Am J Econ Finance. 2022;62:101766. doi: 10.1016/j.najef.2022.101766

[32] Adrian T, Brunnermeier MK. CoVaR. Am Econ Assoc. 2016;106:1705–41.

[33] Caggiano G, Castelnuovo E, Figueres JM. Economic policy uncertainty and unemployment in the United States: a nonlinear approach. Economics Letters. 2017;151:31–4. doi: 10.1016/j.econlet.2016.12.002

[34] Kaya K, Bilgutay NM, Murthy R. Flaw detection in stainless steel samples using wavelet decomposition. In: 1994 Proceedings of IEEE Ultrasonics Symposium, 1994. 1271–4.

[35] Mallat SG. A theory for multiresolution signal decomposition: the wavelet representation. IEEE Trans Pattern Anal Machine Intell. 1989;11(7):674–93. doi: 10.1109/34.192463

[36] Berger T, Gençay R. Improving daily Value-at-Risk forecasts: the relevance of short-run volatility for regulatory quality assessment. J Econ Dynamics Control. 2018;92:30–46. doi: 10.1016/j.jedc.2018.03.016

[37] Trimborn S, Härdle WK. CRIX an index for cryptocurrencies. J Emp Fin. 2018;49:107–22. doi: 10.1016/j.jempfin.2018.08.004

